# Vaccinia Virus Expressing Interferon Regulatory Factor 3 Induces Higher Protective Immune Responses against Lethal Poxvirus Challenge in Atopic Organism

**DOI:** 10.3390/v13101986

**Published:** 2021-10-03

**Authors:** Hana Pilna, Vera Hajkova, Jarmila Knitlova, Jana Liskova, Jana Elsterova, Zora Melkova

**Affiliations:** 1 Department of Immunology and Microbiology, First Faculty of Medicine, Charles University, Studnickova 7, 128 00 Prague 2, Czech Republic; pilna.hana@gmail.com (H.P.); gugpacenpo@seznam.cz (V.H.); jarmila.knitlova@gmail.com (J.K.); janefox@biolog.cz (J.L.); elsterova@paru.cas.cz (J.E.); 2 BIOCEV, Biotechnology and Biomedicine Center of the Academy of Sciences and Charles University in Vestec, Průmyslová 595, 252 50 Vestec, Czech Republic

**Keywords:** IRF-3, vaccinia virus, smallpox, atopic dermatitis, eczema vaccinatum, immunization, interferon beta, interleukin-1 beta, cytokines, Nc/Nga mice

## Abstract

Vaccinia virus (VACV) is an enveloped DNA virus from the Orthopoxvirus family, various strains of which were used in the successful eradication campaign against smallpox. Both original and newer VACV-based replicating vaccines reveal a risk of serious complications in atopic individuals. VACV encodes various factors interfering with host immune responses at multiple levels. In atopic skin, the production of type I interferon is compromised, while VACV specifically inhibits the phosphorylation of the Interferon Regulatory Factor 3 (IRF-3) and expression of interferons. To overcome this block, we generated a recombinant VACV-expressing murine IRF-3 (WR-IRF3) and characterized its effects on virus growth, cytokine expression and apoptosis in tissue cultures and in spontaneously atopic Nc/Nga and control Balb/c mice. Further, we explored the induction of protective immune responses against a lethal dose of wild-type WR, the surrogate of smallpox. We demonstrate that the overexpression of IRF-3 by WR-IRF3 increases the expression of type I interferon, modulates the expression of several cytokines and induces superior protective immune responses against a lethal poxvirus challenge in both Nc/Nga and Balb/c mice. Additionally, the results may be informative for design of other virus-based vaccines or for therapy of different viral infections.

## 1. Introduction

Vaccinia virus (VACV) is an enveloped DNA virus from the Orthopoxvirus family, various strains of which were used in the successful eradication campaign against smallpox in the 1960s and 1970s. Since then, vaccination of the general population was stopped, while different VACV recombinants continue to be used as expression vectors for various laboratory purposes, as well as for experimental preventive and therapeutic vaccines. Nevertheless, after the terrorist attacks on September 11, vaccination of selected professionals was reintroduced, promoting the development of vaccines of the 2nd, 3rd and 4th generations based on purified tissue culture-grown stocks of the NYCBH strain, non-replicating MVA and genetically engineered deletion mutants, respectively [1,2].

Vaccination with the original crude VACV vaccines, as well as that with replicating vaccines of newer generations, reveals a risk of serious, even life-threatening complications. One of them is the development of eczema vaccinatum occurring in individuals suffering atopic dermatitis, excluding them from all preventive vaccination schemes based on replicating VACV [3,4].

Atopic dermatitis (AD) affects approximately 5% of adults and 20% of children in developed countries. There are several types of AD; nevertheless, they all reveal similar features. AD is characterized by increased transepithelial water loss, impaired keratinocyte differentiation, epidermal hyperplasia of skin lesions, increased susceptibility to infections and dysregulated immune responses in general [5,6]. In the context of viral infection, one of the most severe issues is the impaired ability of plasmacytoid dendritic cells, the main producers of type I intereferon (IFN), to immigrate into dermis [7]. Combined with a lower production of antimicrobial peptides, a decreased activity of NK cells and an impaired function of T regulatory cells, this condition can lead to life-threatening complications upon viral infections or vaccination with replicating viruses, namely, eczema herpeticum and eczema vaccinatum [8,9,10,11].

We have previously developed a mouse model of eczema vaccinatum in the spontaneously atopic Nc/Nga mice that reveal many characteristics similar to atopic dermatitis in humans [12,13] and compared it with Balb/c and C57Bl/6 mice, using the wild-type VACV strain WR (wt-WR). We have shown that, after inoculation with wt-WR, Nc/Nga mice developed the largest primary skin lesions and the highest number of satellite lesions, the characteristics of eczema vaccinatum, even without any specific sensitization [4].

VACV is a strong immunogen that simultaneously affects immune responses at multiple levels by encoding a whole range of immune modulatory factors [14]. Particularly, VACV interferes with interferon signaling cascades by inhibiting the expression, activation and action of interferons and interferon-stimulated genes (ISGs). Among others, VACV prevents the phosphorylation and translocation into the nucleus of Interferon regulatory factor 3 (IRF-3), a transcription factor driving the expression of type I interferons [15]. IRF-3 is phosphorylated by TBK1 in response to the recognition of viral dsDNA and dsRNA by several cytoplasmic receptors (PRRs). These cascades are inhibited by VACV gene products E3, A46, K7, C16 and C6. Further, the N2 VACV gene product prevents the transcription function of the phosphorylated IRF-3 in the nucleus [16,17]. VACV gene products also affect signaling cascades leading to the activation of NF-κB, resulting in an altered expression of various cytokines and other factors; further, they prevent posttranslational processing, e.g., processing of IL-1β in inflammasome, or modulate the proteolytic activation and activity of caspases [14,18,19,20,21].

To overcome the VACV-mediated block of IRF-3, we generated a recombinant VACV, strain WR, expressing murine IRF-3 (WR-IRF3) and characterized its effects in tissue cultures, as well as in atopic Nc/Nga and control Balb/c mice. The results presented here demonstrate that the overexpression of murine IRF-3 by VACV results in increased levels of IFNβ and IL-1 β mRNA in tissue cultures, changes in cytokine expression profile in Nc/Nga and Balb/c mice and induction of higher protective immunity against a lethal poxvirus infection.

## 2. Materials and Methods

### 2.1. Ethics Statement

The experiments with mice were carried out in accordance with European regulations for transport, housing and care of laboratory animals (Directive 2010/63/EU on the protection of animals used for scientific purposes). The animals were housed in housing facilities accredited by the Ministry of Agriculture of the Czech Republic and monitored daily. All the infectious experiments were performed in the Biosafety Level 2 laboratory with negative pressure and using HEPA-filtered animal boxes. Full details of the animal experiments, including the mortality aspects, were approved by the Experimental Animal Use Committee of the 1st Medical Faculty of Charles University and the Ministry of Education of the Czech Republic (experimental protocols No. GA UK 100307 – 208/06, 0021620806 MSM, P302/10/0083 – 298/09). These protocols also addressed the cases of unexpected mortality. All individuals working with infected animals had been vaccinated with the standard smallpox vaccine in the past.

### 2.2. Chemicals

All the media and growth supplements and other chemicals were purchased from Gibco and Sigma, unless otherwise specified.

### 2.3. Cells

African green monkey kidney epithelial cell line BSC-40 (ATCC, CRL-2761), mouse embryonal fibroblasts NIH 3T3 (ATCC, CRL-1658) and mouse monocyte/macrophage cells J774.G8 (kindly provided by Dr. Josef Michl, State University of New York, Health Science Center at Brooklyn, USA) were used. The cells were grown in DMEM with addition of 10% NCS (BSC-40) or FBS (NIH 3T3, J774.G8) and antibiotics as described previously [22,23]. 

### 2.4. Viruses

The VACV strain Western Reserve (WR) and VACV recombinants derived from the wild-type VACV (wt-WR), prepared by a homologous recombination into the wt-WR thimidine kinase gene, were used. VACV recombinant expressing luciferase under control of a VACV early/late promoter p7.5 (WR-Luc) was described previously [24]. The recombinant VACV expressing mouse IRF-3 under control of p7.5 (WR-IRF3) was generated using IRF-3 cDNA obtained by reverse transcription and PCR amplification of RNA isolated from NIH 3T3 cells. The product was inserted into the pcDNA3 plasmid (Clontech, Mountain View, CA, USA), then sequenced and subcloned into the pSC11 insertion vector [25]. This vector was used for a homologous recombination into the thymidine kinase gene of wt-VACV genome. The recombinant WR-IRF3 was selected in a way described previously [24]. The insertion was verified with Southern blotting using a VACV HindIIIJ fragment and IRF-3 cDNA as probes.

The viruses were propagated in BSC-40 cells and their titers were determined by serial dilutions and plaque assays in BSC-40 cells as described previously [26]. Crude stocks of the recombinants were used in BSC-40 and NIH 3T3 cells, while sucrose gradient-purified viruses [27] were used in J774.G8 macrophages and in mice. For experiments, 0.6 × 10^6^ cells in a 24-well plate or 1.1 × 10^6^ of cells in a 12-well plate were infected at a multiplicity of infection (M.O.I.) specified for each experiment for 1 h. Then, 2% NCS-DMEM or 2% FBS-DMEM was added and the cells were incubated for the indicated periods of time (hours post infection, h.p.i.).

### 2.5. Animals and Infection

Male and female mice of strains Nc/Nga (kind gift from Riken BioResource center, Japan) and Balb/c were bred and reared in specific pathogen-free (SPF) conditions in The Center for Experimental Biomodels at the 1st Medical Faculty, Charles University. The faculty facility ensured standard SPF conditions (HEPA-filtered air, sterilized beddings and food, all manipulations in laminar flow hoods) and routine veterinary check-ups. The animals were acclimatized in conventional housing for at least 1 week before the start of experiments at the age of 6–8 weeks. The animals were assigned to cages and/or experimental groups based on their sex and litter with a maximum of 5 mice/cage.

All the experimental manipulations with mice were performed in anesthesia with avertin (2,2,2,tribromoethanol in tertial amylalcohol, 1 mg/mL) administered intra-peritoneally (i.p.; 12–16 µL/g of weight). For the induction of the protective immunity, the mice were inoculated either transdermally (t.d.; volume of inoculum, 1 µL) using acupuncture needles HuanQiu Super (Suzhou Huanqiu Acupuncture Medical Appliance, Suzhou, China) or intranasally (i.n.; volume of inoculum, 5 µL) with different doses of WR-Luc or WR-IRF3. Twenty-four days later, they were infected i.n. with a lethal dose of wt-WR, the surrogate of smallpox (10^5^ PFU/mouse; volume of inoculum, 30 µL) [4]. The mice in the experiments were monitored daily for weight, body temperature, fur and behavioral changes. The criteria for humane endpoint defined in the study protocol P302/10/0083 – 298/09 were weight loss under 25% and decreased body temperature. In case the mice did not succumb to the infection and survived, they were euthanized 12–15 days post infection (p.i.). All efforts were made to minimize the suffering of the mice during manipulations with them.

### 2.6. Determination of Virus Titer

Tissue culture samples were collected by pipetting in culture media and processed in two freeze-thaw cycles. The skin lesions of infected animals were frozen and homogenized in ice-cold DMEM in a volume corresponding to 4 times the weight of the skin. Consequently, the samples were thawed and frozen twice, sonicated and centrifuged. The whole tissue culture samples or supernatants of infected skin samples were used for determination of virus titer using serial dilutions and plaque assays in BSC-40 cells as described previously [26].

### 2.7. Determination of mRNA Levels

Cell or tissue samples were homogenized in RNA Blue (Top-Bio, Prague, Czech Republic) and total RNA was isolated according to manufacturer’s protocol. The RNA concentration and purity were determined by measuring the absorbance at 260 and 280 nm, respectively, using a UV spectrophotometer BioPhotometer (Eppendorf AG, Hamburg, Germany). RNA was then treated with DNase (Fermentas, Vilnius, Lithuania) and used for one-step RT-qPCR using a SensiFAST™ SYBR^®^ Hi-ROX One-Step Kit (Bioline, London, UK) and Applied Biosystems 7300 Real-time PCR System (Applied Biosystems, Waltham, MA, USA), according to manufacturer’s protocol, employing gene-specific primers and universal cycling conditions [4]. Primers for the cytokines and other factors were designed using Primer-BLAST or adopted from the literature. Sequences of individual primer pairs are listed in Table 1. The mean of technical duplicates was used for relative quantification of each target compared to GAPDH. Additionally, total RNA was reverse-transcribed and used for conventional PCR.

### 2.8. Western Blot Analysis

A western blot analysis was performed as described previously [18]. Cell lysates were resolved by SDS-PAGE and transferred to a low-fluorescence PVDF membrane (Azure Biosystems, Dublin, CA, USA). Individual proteins were labelled with specific antibodies and visualized by chemiluminescence and SuperSignal^®^ West Femto Maximum Sensitivity Substrate (Thermo Scientific, Waltham, MA, USA) or by near infrared fluorescence using Azure Biosystems c600. IRF-3 was detected with a mouse monoclonal antibody (BioLegend, San Diego, CA, USA, dilution 1:1,000) and a peroxidase-conjugated goat anti-mouse antibody (Sigma Co., St. Louis, MO, USA, dilution 1:10,000); phosphorylated IRF-3 was detected with a rabbit monoclonal antibody (Cell Signaling Technology, Danvers, MA, USA) and a IR 700-conjugated goat anti-rabbit antibody (Azure Biosystems, Dublin, CA, USA, dilution 1:10,000); β-actin was detected with a rabbit polyclonal antibody (Abcam, Cambridge, UK, dilution 1:2,500) and peroxidase-conjugated goat anti-rabbit antibody (MP Biomedicals-Cappel, Solon, OH, USA, dilution 1:10,000); PARP-1 was detected with a rabbit polyclonal antibody (Santa Cruz Biotechnology, Dallas, TX, USA, dilution 1:500) and peroxidase-conjugated goat anti-rabbit antibody (MP Biomedicals-Cappel, Solon, OH, USA, dilution 1:10,000).

### 2.9. Fluorescence Microscopy

Cultured cells were visualized in situ in tissue culture plates using an inverted fluorescent microscope Olympus IX70 equipped with a CCD camera ProgRes MFcool (Jenoptik AG) and NIS-Elements BR3.1 software (Laboratory Imaging Ltd., Prague, Czech Republic). The cells were observed in phase contrast and under UV light after addition of Hoechst 33,342 (Intergen, Purchase, NY, USA; final concentration, 0.2 µg/mL) [21]. All pictures were captured at original magnification 150×.

### 2.10. Statistical Analyses

Statistical analyses were performed using GaphPad and one or two-tailed unpaired Student’s *t*-tests, with statistical significance at ^x^ *p* < 0.1, * *p* < 0.05 and ** *p* < 0.01. The results are presented as means +/− SEM (standard error of mean) or as GM *÷ GM SD (geometrical means with geometric standard deviation). The survival of mice was analyzed using the log-rank Mantel–Cox test.

## 3. Results

### 3.1. Expression of Mouse Recombinant IRF-3 in Cell Lines and Its Effects

#### 3.1.1. Expression of IRF-3 by WR-IRF3

**Figure 1:**
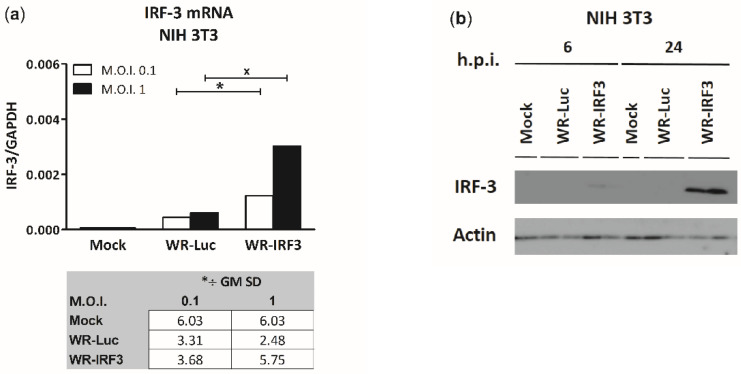
Expression of mouse IRF-3 by WR-IRF3 in NIH 3T3 fibroblast cell line. The cells were mock-infected or infected with WR-Luc or WR-IRF3 at M.O.I. of 0.1 and 1. At individual time points, the cells were collected and used for further analysis. (**a**) 1-step RT-qPCR analysis at 24 h.p.i. IRF-3 mRNA was normalized to GAPDH mRNA and expressed as GM *÷ GM SD; n = 4. Statistically significant differences at ^x^ *p* < 0.1, * *p* < 0.05 (one-tailed unpaired Student’s *t*-test). (**b**) Western blot analysis. M.O.I. of 1. The cell lysates were resolved by 10 % SDS-PAGE. IRF-3 and control β-actin were detected by western blot analysis and chemiluminescence. Mock, mock-infected cells; WR-Luc, cells infected with VACV expressing luciferase; WR-IRF3, cells infected with VACV expressing Mu IRF-3.

The expression of mouse IRF-3 by WR-IRF3 was confirmed in mouse embryonal fibroblasts NIH 3T3 first at the level of mRNA using a 1-step RT-qPCR (Figure 1a) and then at protein level using a western blot analysis (Figure 1b). In cells infected with control vaccinia virus expressing luciferase, WR-Luc, no endogenous IRF-3 protein was detected, probably due to its much lower levels. Similar results were also obtained in mouse monocyte/macrophage cells J774.G8 (Figure S1). The attempt to detect the phosphorylated form of IRF-3 was unsuccessful.

#### 3.1.2. Growth of WR-IRF3 Is Increased in Mouse Cells

**Figure 2:**
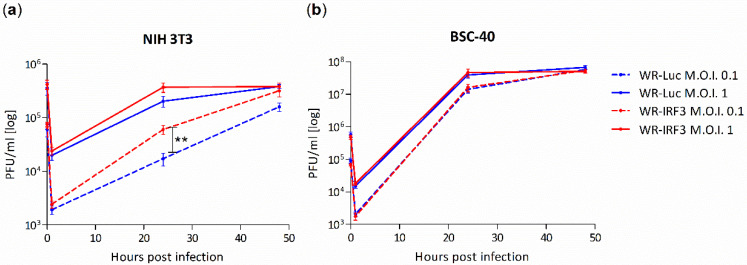
IRF-3 expressed by WR-IRF3 increases VACV growth in mouse cells. (**a**) NIH 3T3 and (**b**) BSC-40 cells were infected with WR-Luc or WR-IRF3 at M.O.I. of 0.1 and 1. At individual time points, the cells were collected and used for determination of virus titer. Graphs represent means of 3 independent experiments performed in duplicates +/− SEM. ** *p* < 0.01 (two-tailed unpaired Student’s *t*-test). WR-Luc, cells infected with VACV expressing luciferase; WR-IRF3, cells infected with VACV expressing Mu IRF-3.

The effect of the expression of the mouse IRF-3 on VACV growth was characterized in mouse fibroblasts NIH 3T3, as well as in African green monkey kidney epithelial cells BSC-40 (Figure 2). Contrary to our expectations, the growth of WR-IRF3 was consistently higher than the one of WR-Luc in mouse NIH 3T3 cells. On the other hand, there were no differences in the growth of the two viruses in heterologous BSC-40 cells, confirming the species specificity of this transcription factor.

#### 3.1.3. WR-IRF3 Induces Increased Levels of IFNβ and IL-1β mRNA

**Figure 3:**
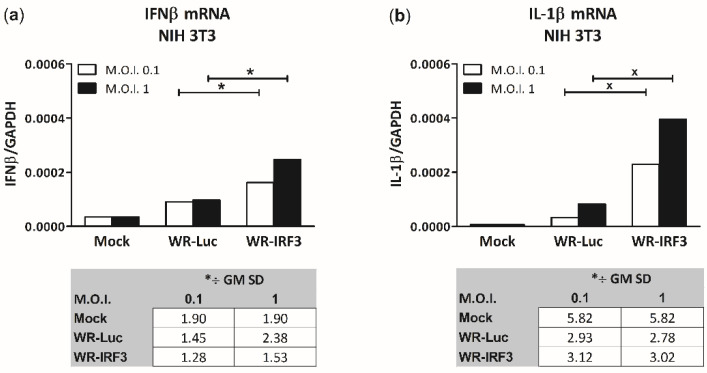
Infection with WR-IRF3 increases mRNA levels of (**a**) IFNβ and (**b**) IL-1β in NIH 3T3 fibroblasts. The cells were mock-infected or infected with WR-Luc or WR-IRF3 at M.O.I. of 0.1 and 1. At 24 h.p.i., the cells were collected and used for 1-step RT-qPCR analysis. IFNβ and IL-1β mRNA was normalized to GAPDH mRNA and expressed as GM *÷ SD; n = 4. Statistically significant differences at ^x^ *p* < 0.1, * *p* < 0.05 (one-tailed unpaired Student’s *t*-test). Mock, mock-infected cells; WR-Luc, cells infected with VACV expressing luciferase; WR-IRF3, cells infected with VACV expressing Mu IRF-3.

VACV encodes several genes preventing phosphorylation of IRF-3 and its translocation into the nucleus [15], thus inhibiting expression of its target genes, namely, IFNβ. Therefore, we designed a functional assay assessing the effect of IRF-3 overexpressed by WR-IRF3 on the IFNβ expression. We also chose to characterize IL-1β as a gene target expressed independently, by the action of NF-κB [29]. In NIH 3T3 fibroblasts, WR-IRF3 induced a moderate increase in mRNA levels of IFNβ, compared to WR-Luc (approx. 2-fold; Figure 3a), while it unexpectedly induced a high increase in mRNA levels of IL-1β (5–7-fold, compared to WR-Luc) (Figure 3b). In agreement with previous experiments, there was no effect of IRF-3 on the IFNβ expression in heterologous BSC-40 cells (data not shown). On the other hand, in J774.G8 monocyte/macrophage cells, infection with WR-Luc induced a decrease in the levels of IL-1β mRNA (to 20% of mock-infected cells), while WR-IRF3 preserved them (Figure S2).

#### 3.1.4. WR-IRF3 Induces Increased Apoptosis in Mouse Cells

**Figure 4:**
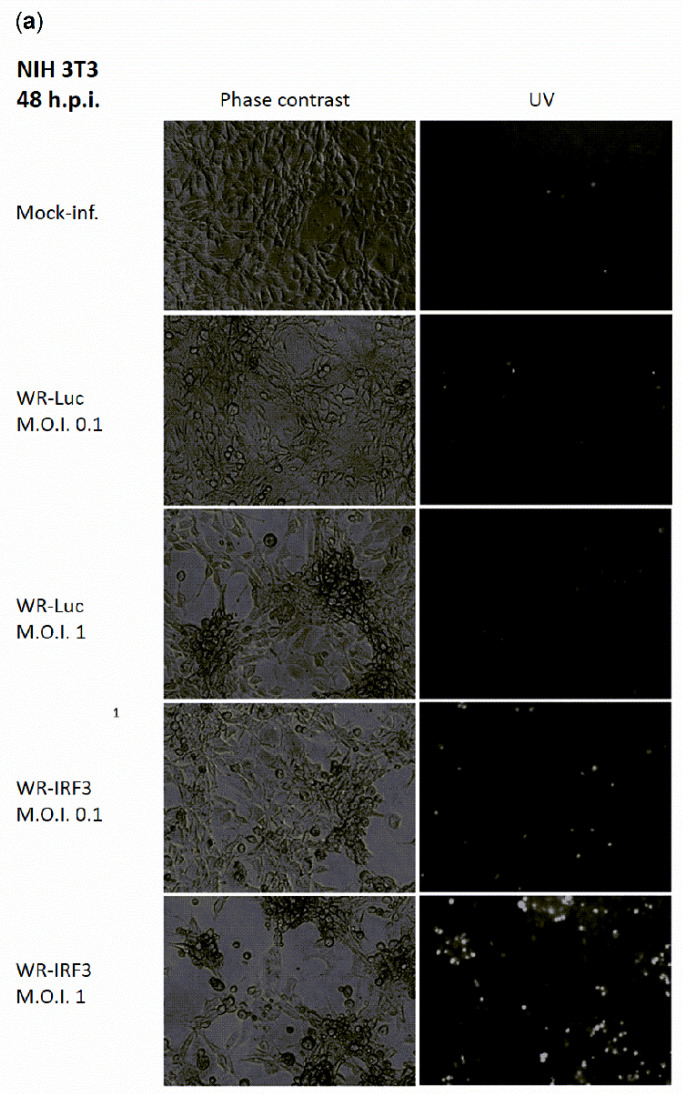

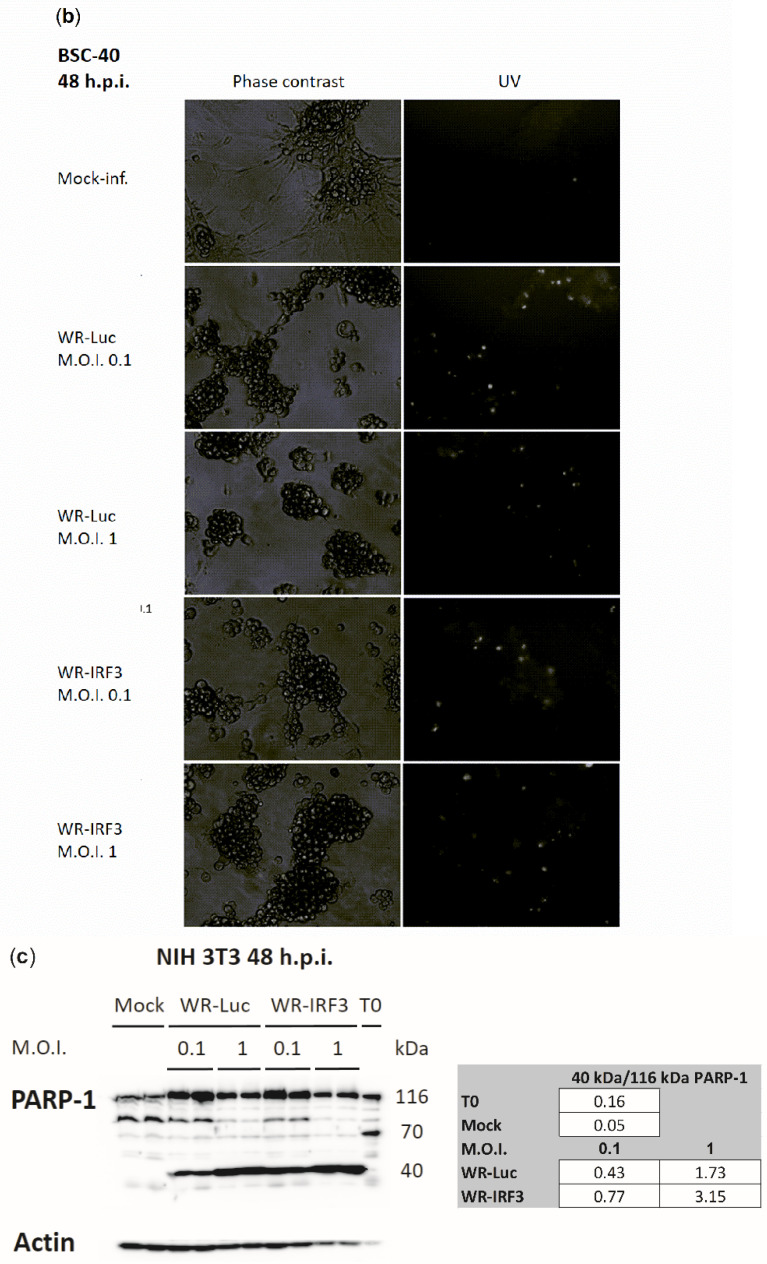
WR-IRF3-infected NIH 3T3 cells undergo apoptosis. NIH 3T3 and BSC-40 cells were mock-infected or infected with WR-Luc or WR-IRF3 at M.O.I. of 0.1 or 1. (**a**,**b**) Microscopy. At 48 h.p.i., Hoechst 33342 was added to the culture media and the phase contrast and UV-fluorescent pictures were captured at original magnification 150×. (**c**) PARP-1 cleavage in VACV-infected cells. At 48 h.p.i., the cells were collected and the cell lysates were resolved by 8% SDS-PAGE. PARP-1 cleavage and control β-actin were detected by western blot analysis and chemiluminescence. Mock, mock-infected cells; WR-Luc, cells infected with VACV expressing luciferase; WR-IRF3, cells infected with VACV expressing Mu IRF-3; T0, time of infection.

It has been previously demonstrated that the overexpression of IRF-3 results in apoptosis [30]. Accordingly, we have observed increased apoptosis of WR-IRF3-infected mouse NIH 3T3 cells at 48 h.p.i., as characterized by optical and fluorescence microscopy using phase contrast and Hoechst 33342 staining of the condensed heterochromatin, respectively (Figure 4a). On the other hand, in heterologous BSC-40 cells, the effect of infection with both viruses was comparable (Figure 4b). In NIH 3T3 fibroblasts, the extent of apoptosis was further characterized by levels of PARP-1 cleavage determined by western blotting and chemiluminescence (Figure 4c). At 48 h.p.i., the ratio of the 40 kDa cleavage product versus 116 kDa full-length PARP-1 was found to be approximately 2-fold higher in WR-IRF3-infected cells, compared to WR-Luc-infected cells, as determined by the quantification of the chemiluminescent signal.

### 3.2. Experiments with Mice

Atopic individuals are excluded from all smallpox preventive vaccination schemes based on replicating vaccinia viruses because of the risk of development of eczema vaccinatum, a life-threatening complication characterized by the inadvertent spread of virus [3]. In order to assess the properties of WR-IRF3 as a vaccination vector, we used spontaneously atopic Nc/Nga mice in comparison with control, Th2-skewed Balb/c mice [4].

#### 3.2.1. Growth of WR-IRF3 in Mice and Its Effects

**Figure 5:**
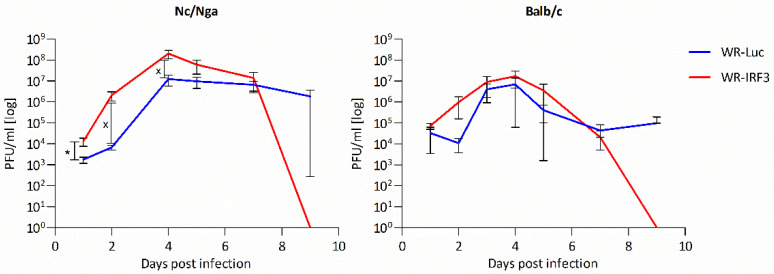
Growth of WR-IRF3 is increased in mouse skin lesions. Nc/Nga and Balb/c mice were transdermally inoculated with 10^4^ PFU of a purified stock of WR-Luc or WR-IRF3 and sacrificed at indicated days p.i. Skin biopsies were taken and virus titers (PFU/mL) in the lesions were determined. Graphs represent mean +/− S.E.M., n = 3 mice/group. Statistically significant differences at * *p* < 0.05, ^x^ *p* < 0.1 (one-tailed unpaired Student’s *t*-test). WR-Luc, mice infected with VACV expressing luciferase; WR-IRF3, mice infected with VACV expressing Mu IRF-3.

First, the mice were transdermally (t.d.) inoculated with WR-Luc and WR-IRF3 and the kinetics of virus growth was determined (Figure 5). Similarly to the growth in NIH 3T3 fibroblasts, WR-IRF3 reached somewhat higher titers than WR-Luc in skins of both mouse strains. In Nc/Nga mice, both viruses reached higher titers and WR-IRF3 induced skin lesions that were, in general, larger than those induced by WR-Luc. Nevertheless, in both mouse strains, WR-IRF3-induced lesions resolved faster and no infectious virus was detected by day 9 p.i.

Additionally, the weight of spleens in Nc/Nga mice t.d. inoculated with WR-IRF3 were found increased when normalized to the weight of mice. In mice inoculated intranasally (i.n.), the weight of spleens was not found consistently changed. On the other hand, levels of IL-1β mRNA in spleens of mice i.n. infected with WR-Luc were found decreased, while WR-IRF3 supported their higher levels (Figure S3).

#### 3.2.2. WR-IRF3 Induces Higher Protective Immunity against Lethal Poxvirus Challenge

**Figure 6:**
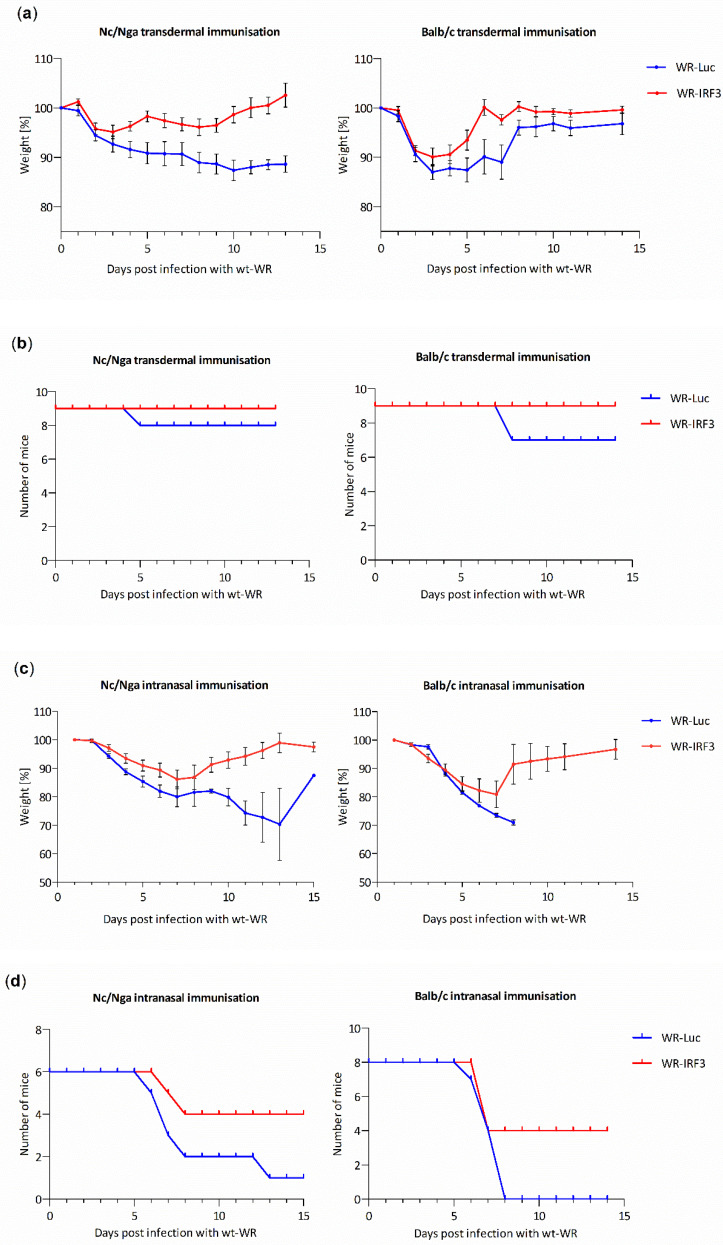
Immunization with WR-IRF3 induces better protective immunity against a lethal challenge with wt-WR. Nc/Nga and Balb/c mice were immunized t.d. (10^4^ PFU/mouse) or i.n. (10^3^ PFU/mouse) with purified WR-Luc or WR-IRF3. At 24 days later (day 0 in the graphs), they were infected i.n. with a lethal dose of wt-WR (10^5^ PFU/mouse). Changes in body weight and survival of mice immunized (**a**,**b**) t.d. and (**c**,**d**) i.n. are presented. (**a**,**c**) Graphs represent means +/− S.E.M. The survival of mice of both strains immunized i.n. with WR-IRF3 (**d**) was statistically significant at *p* < 0.1 (log-rank Mantel–Cox test). Statistically significantly higher body weight of mice immunized with WR-IRF3 (one-tailed unpaired Student’s *t*-test): (**a**) Nc/Nga t.d. immunization, 1 and 2 days p.i., *p* < 0.1; 4–13 days p.i., *p* < 0.05; Balb/c t.d. immunization, 9–10 days p.i., *p* < 0.1; 5–8, 11 days p.i., *p* < 0.05; (**c**) Nc/Nga i.n. immunization, 2–5, 8–12 days p.i., *p* < 0.05; Balb/c i.n. immunization, 6 days p.i., *p* < 0.1; 2 and 7 days p.i., *p* < 0.05. WR-Luc, mice immunized with VACV expressing luciferase; WR-IRF3, mice immunized with VACV expressing Mu IRF-3.

The ability of WR-IRF3 and WR-Luc to induce protective immunity against a lethal infection with wild-type VACV strain WR, the surrogate of smallpox, was assessed in both mouse strains. Mice were immunized t.d. or i.n. with different doses of the two viruses and, 24 days later, they were i.n. infected with a lethal dose of wt-WR. The graphs presented in Figure 6 summarize weight loss and survival of Nc/Nga and Balb/c mice immunized with the lowest effective doses used for t.d. and i.n. immunization, i.e., with 10^4^ and 10^3^ PFU/mouse, respectively. The results indicate that the immunization with WR-IRF3 at these doses provides a more efficient protective immunity against a lethal poxviral infection in both atopic Nc/Nga and control Balb/c mice. Nevertheless, i.n. immunization of Balb/c mice with a higher dose, 10^4^ PFU/mouse, induced, in response to the lethal challenge, a greater weight loss and even death of animals immunized with WR-IRF3, while the weight changes were comparable in Nc/Nga mice (Figure S4). Intranasal and t.d. immunizations with the two VACV recombinants at the dose of 10^5^ PFU/mouse provided comparable protection in mice of both strains (data not shown).

#### 3.2.3. Changes in Interferon and Cytokine mRNA Levels in Mouse Skin Lesions

In order to obtain more insight into the mechanism underlying the induction of the better protective immunity by WR-IRF3, we analyzed the changes in the levels of selected cytokines in skin lesions after the t.d. inoculation of WR-IRF3 and WR-Luc, in comparison with lesions after mock-infection with PBS. We determined the mRNA levels of IFNα, β—the primary antiviral interferons expressed by the action of IRF-3, IL-1β and TNF-α—the cytokines initializing the inflammation, Th1/Th2/Th17 signature cytokines, IFNγ-stimulated AIM2 and iNOS, and IL-10 and TGFβ1, 3—the cytokines important for the resolution of the inflammation.

Before the inoculation, the levels of all the mRNAs studied were comparable between Nc/Nga and Balb/c mice. Mock-infection with PBS resulted in aseptic wound-healing, while this pattern changed in VACV-infected lesions depending on the VACV recombinant and the mouse strain used.

**Figure 7:**
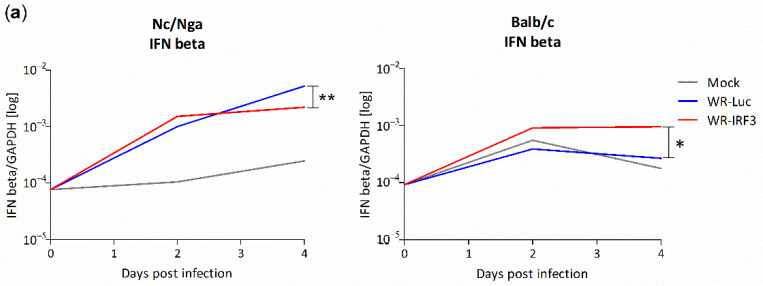

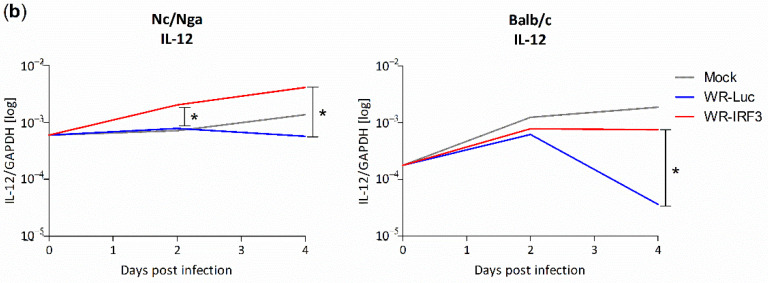
Changes in IFNβ and IL-12 mRNA levels in mouse skin lesions. Nc/Nga and Balb/c mice were t.d. inoculated with PBS or 7.5 × 10^5^ PFU/mouse of purified WR-Luc or WR-IRF3, sacrificed at indicated days p.i. and RNA from skin lesions was used for 1-step RT-qPCR analysis. (**a**) IFNβ and (**b**) IL-12 mRNA was normalized to GAPDH mRNA. Graphs represent geometrical means, n = 3 mice/group. Statistically significant differences at * *p* < 0.05, ** *p* < 0.01 (two-tailed unpaired Student’s *t*-test). See Figure S5 for further details.

In mock-infected Nc/Nga mice, a marked increase in IFNα mRNA levels was observed at both day 2 and 4 p.i. (10- and 70-fold, respectively), while moderate but continuous increases were observed in the mRNA levels of IFNβ, IFNγ, IL-12 and IL-17A. An increase at 2 days p.i. followed by a decrease or a stagnation at 4 days p.i. were found for iNOS, IL-1β, AIM2, TNFα, IL-4, IL-10 and TGFβ1; TGFβ3 mRNA levels practically did not change. Upon inoculation with WR-Luc, the levels of most mRNAs, except of IL-12, were found increased up to day 4 p.i. (more than 10-fold, compared to mock-infected mice; Figure S5). The biggest increases were observed in mRNA levels of IFNγ (together with iNOS; both 60-fold increases) and IL-4 (100-fold), the key cytokines for the Th1 and Th2 polarization, respectively. IRF-3 should induce expression of type I IFNs; however, compared to WR-Luc, the inoculation of WR-IRF3 induced only a limited increase in IFNβ (Figure 7a) and IFNα mRNA levels (Figure S5a) at 2 days p.i., while their total levels revealed a stagnation at day 4 p.i. With the exception of IL-12, upon infection with WR-IRF3, the levels of other mRNAs tended to decrease or normalize back to the levels found in mock-inoculated Nc/Nga mice within 4 days p.i. (Figure 7b and S5).

In Balb/c mice, levels of most mRNAs studied increased in mock-infected mice at 2 days p.i., followed by a stagnation or a decrease at 4 days p.i. The highest initial increase was observed in mRNA levels of IL-1β, TNFα and IL-4 (66-, 39- and 41-fold, respectively). The inoculation with WR-Luc did not induce any substantial changes, compared to mock-infected samples, in the mRNA levels of IFNβ, AIM2, IL-1β, IL-17A and IL-10, while the levels of IFNα and γ mRNAs were higher and the levels of iNOS, TNFα, IL-12, TGF-β1 and 3 lower. The levels of IL-4 mRNA reached lower values in WR-Luc than in mock-infected samples at 2 days p.i., but revealed quite a strong increase at 4 days p.i. (150-fold higher than mock-infected samples). Compared to WR-Luc, the inoculation with WR-IRF3 resulted in a 2–3-fold increase in the levels of IFNβ mRNA at both time points (Figure 7a). Most other mRNAs were relatively comparable to WR-Luc at 2 days p.i., but revealed an increase at 4 days p.i. Only the levels of TGF-β3 mRNA were found decreased at both time points (Figure S5).

Altogether, the only cytokine mRNA found increased in both mouse strains at both time points in response to WR-IRF3, compared to WR-Luc, was IL-12 (Figure 7b). Possibly, this increase might underlie the better protective immunity induced by WR-IRF3 in both mouse strains [31].

## 4. Discussion

In this work, we describe the generation and characterization of a VACV recombinant expressing IRF-3 that was initially prepared in the attempt to design a safer vaccine against smallpox. Today, a non-replicating vaccine based on MVA is available for human use, demonstrating satisfactory safety and immunogenicity [32,33,34]. An attenuated replication-competent third-generation vaccine LC16m8 derived from the VACV strain Lister is also available, but it may still impose a risk for immunocompromised or atopic individuals [35,36]. The results presented in this paper bring new data describing the IRF-3-mediated modulation of VACV–host interactions that could be useful in the design of other virus-based vaccines or in the treatment of different viral infections. IRF-3 is a key transcription factor of type I IFNs, the first-line innate immunity agents that play a critical role in the induction of specific immunity and control of virus growth. Consequently, the functions of IRF-3, as well as the signaling cascades leading to its activation by STING and TBK1, are actively targeted and inhibited by many viruses, e.g., by poxviruses, herpes viruses or adenoviruses, hepatitis-inducing viruses, such as HBV, HCV or HAV, BDV, but also by Ebola, Dengue, Zika and the lately emerged SARS-CoV-2 [37,38,39].

Endogenous IRF-3 is constitutively expressed in the inactive form in most cell types. Upon phosphorylation by TBK1, dimerization and nuclear translocation, it drives the expression of several types of IFNs and ISGs that play key roles in the response to viral infection, its control and elimination [40]. These and other immune responses are efficiently controlled and counteracted by a variety of VACV-encoded gene products, leading to the fine tuning of virus–host interactions and resulting in the induction of a powerful protective immunity accompanied by containment of the virus. Downstream in the IRF-3 signaling cascade, the VACV-encoded C6 protein inhibits the kinase activity of TBK1 that is activated in response to various PRRs, while N2 directly prevents the transcription mediated by phosphorylated IRF-3 in the nucleus. Other VACV-encoded proteins, such as K7 or A49, inhibit the transcription activity of NF-κB, that is activated upon phosphorylation of IκB mediated by TBK1 and other IKKs, targeting IκB for degradation [16,17,19,20]. In predisposed individuals, this intricate balance can be disturbed, facilitating virus replication and promoting the development of serious complications. In atopic individuals, the immigration of pDCs, the main producers of IFNα, as well as the function of other immune cell types critical for virus control, is compromised [5,7,41]. Thus, we hypothesized that promoting the expression of IRF-3 and type I IFNs should be beneficial.

In the first part of the study, we confirmed the expression of a functional murine IRF-3 by WR-IRF3 that was able to stimulate an increase in the levels of IFNβ mRNA, in comparison with WR-Luc, in mouse cell lines. Yet, we were unable to detect the phosphorylated form of IRF-3 that should mediate the IFNβ transcription. In mouse skin lesions, WR-IRF3 induced a moderate increase in the levels of IFNβ and IFNα mRNA, in comparison with WR-Luc. These results indicate that the VACV-induced block of IRF-3-mediated responses was overcome by VACV expressing IRF-3. Nevertheless, NF-κB and ATF-2/c-Jun also participate in the transcription of IFNβ [42,43,44].

Along these lines, upon infection with WR-IRF3, we observed changes in mRNA levels of IL-1β, the transcription of which is mediated by NF-κB [29,45]. In NIH 3T3 fibroblasts infected with WR-IRF3, a marked increase in IL-1β mRNA, compared to WR-Luc, was found. In the infected J774.G8 macrophages, as well as in spleens of VACV inoculated mice, the infection with WR-Luc induced a decrease in IL-1β mRNA levels, while WR-IRF3 preserved them. Finally, in mouse skin lesions, WR-IRF3 further increased IL-1β mRNA levels in Balb/c mice, while it returned them closer to the levels of mock-infected controls in atopic Nc/Nga mice. There are no reports suggesting a direct interaction of IRF-3 with the IL-1β promoter, but both IκB and IRF-3 are phosphorylated by TBK1 in response to STING activation by different cytoplasmic dsDNA and dsRNA sensors [46,47,48,49]. Thus, it is possible to speculate that, in the context of a VACV infection, the overexpression of IRF-3 might shift the signaling through these overlapping transduction pathways. In contrast to our findings, there are reports indicating that type I IFNs mediated a decrease in the expression of IL-1β and other pro-inflammatory cytokines [50,51]. Similar effects were also observed upon expression of the exogenous IRF-3 [52]. Indeed, we did not observe any increase in IL-1β mRNA levels upon transfection of IRF-3 expressing plasmid either (unpublished results). Possibly, the increase in IL-1β mRNA levels induced by IRF-3 expression is mediated indirectly and only upon infection with a cytoplasmic DNA virus VACV, as suggested by the modest stimulatory effects of WR-Luc. Nevertheless, changes in mRNA levels may not directly translate into changes in protein levels. In addition, the production of the functional IL-1β is regulated mainly at the post-translational level in inflammasome through the action of caspase-1 and AIM-2, which is modulated by VACV-encoded SPI-2 and F1L [53,54,55,56].

Interferons were originally discovered based on their ability to interfere with virus growth [57]. In most tissue cultures, VACV is resistant to type I IFN, as it encodes many inhibitors preventing the induction of the expression or activation of ISGs, e.g., dsRNA-responsive 2′5′OAS/RNaseL or PKR [58,59]. Nevertheless, in vivo, VACV replication is sensitive to administration of type I IFNs [60,61]. The overexpression of IRF-3 by WR-IRF3 resulting in the increased expression of IFNβ would be expected to improve the antiviral state of the neighboring cells and to decrease VACV replication. Yet, in comparison with WR-Luc, the growth of WR-IRF3 was reproducibly higher in mouse cells, whereas no differences were found in heterologous BSC-40 cells. Likewise, the growth of this recombinant was higher in the skin lesions of both mouse strains. It is hard to speculate about the reasons of the increased WR-IRF3 growth. Possibly, the induction of apoptosis might be energetically more favorable to VACV replication than the abrupt lytic, i.e. necrotic type of cell death. Increased apoptosis could be also the underlying cause of larger skin lesions induced by WR-IRF3, while it might contribute to the more efficient protective immunity observed in response to WR-IRF3, as the pathogen-induced apoptosis accompanied by the formation of various PAMPS and DAMPS can be more immunogenic [62,63]. The improved immune responses, namely the increased expression of IL-12, probably also contribute to the faster resolution of lesions induced by WR-IRF3 than by WR-Luc (Figure 5), or wt-WR (unpublished results). Such faster clearance of the virus is likely to be beneficial to avoid complications in atopic individuals, as well as to decrease the risk of virus transmission by vaccinees from the general population.

VACV is considered as a prototype lytic virus, but it has been demonstrated to induce apoptosis in distinct cell types, as well as in mouse skin lesions [4,18,64,65,66]. The increased expression of IRF-3 has also been previously shown to induce apoptosis [67]. IRF-3 can bind the pro-apoptotic Bax, translocate into the outer mitochondrial membrane and initiate the intrinsic apoptotic pathway [30]. Additionally, an increased production of type I IFNs due to IRF-3 can activate the extrinsic apoptotic pathway [68].

Vaccines based on replicating VACV are typically administered by pricks in the skin with a bifurcated needle. Nevertheless, i.n. administration usually induces a better mucosal immunity against air-born infections. When comparing the two ways of immunization, i.n. immunization with WR-IRF3 provided a more efficient protective immunity against a lethal wt-WR challenge, in comparison with WR-Luc, at lower dose than the t.d. immunization (i.e., 10^3^ PFU/mouse i.n. compared to 10^4^ PFU/mouse t.d.) in both mouse strains. Yet, i.n. immunization with a higher dose of WR-IRF3 (10^4^ PFU/mouse) induced a bigger weight loss than WR-Luc in Balb/c mice, suggesting complicated dose-dependent responses in the individual mouse strains.

In order to obtain more insight into the mechanism of the induction of the higher protective immunity by WR-IRF3, we analyzed mRNA levels of the type I IFNs, Th1/Th2/Th17 signature cytokines, AIM2 and iNOS in the skin of Nc/Nga and Balb/c mice. Despite the fact that the replication kinetics of both viruses and the character of protective immunity were relatively comparable between the two mouse strains, the pattern of most cytokine mRNA changes was different. However, the changes in mRNA levels may not correlate with the production of the functional proteins.

The mock-infection with PBS resulted in changes characterizing the aseptic wound-healing leading to resolution, while this pattern was changed in VACV-infected lesions depending on the VACV recombinant and the mouse strain. In mock-infected, as well as in WR-Luc-infected mice of both strains, the highest increases were observed in IL-4 mRNA levels, correlating with the Th2-skewed acute immune responses [69]. The WR-Luc-induced increase was down-modulated by WR-IRF3 in Nc/Nga mice, in agreement with the general effects of this recombinant on mRNA levels of other cytokines and factors in this strain. In Nc/Nga mouse skin infected with WR-Luc, a high increase was also observed for IFNα, γ and iNOS, consistent with the chronic inflammation occurring in the atopic skin. In contrast, in Balb/c mice, an initially high increase was observed for IL-1β and TNFα, cytokines that are important in the early phase of inflammation. In this strain, the WR-IRF3 infection generally tended to increase mRNA levels of most cytokines and factors. The only cytokine mRNA found increased by WR-IRF3 compared to WR-Luc in both mouse strains at both time points was IL-12, one of the key cytokines determining Th1 polarization and playing a vital role in antiviral responses [70]. Thus, this increase could correspond to the faster resolution of WR-IRF3-infected skin lesions, as suggested by the results with VACV recombinant expressing IL-12 [31]. On the other hand, differential increases in IFNγ and iNOS mRNA levels did not seem to correlate with distinct growth patterns of WR-Luc or WR-IRF3 growth in skin lesions of the two strains, despite their previously proven role in the control of VACV replication [71,72,73,74].

IL-17 plays an important role in enhancing antiviral immune responses, but it may also promote and exacerbate virus-induced pathology mediated by distinct mechanisms [75]. In VACV-infected atopic skin, increased levels of IL-17 were described to modulate immune responses against VACV, promote neutrophil infiltration and compromise the function of NK cells, resulting in higher virus growth and replication [8,76,77]. On the level of mRNA, we observed an increase in IL-17A in response to WR-Luc that was decreased by WR-IRF3 in Nc/Nga mouse skin, while the levels induced by WR-Luc were comparable to or lower than those in mock-inoculated skin and increased by WR-IRF3 in Balb/c skin lesions. Nevertheless, in the supernatants of cultured splenocytes prepared from infected mice, we observed a higher increase in IL-17A in response to WR-IRF3 compared to WR-Luc in both mouse strains, as determined by a cytokine bead assay on the protein level (preliminary results).

Susceptibility of the atopic skin to bacterial, namely, staphylococcal, and viral infections is due to compromised, dysregulated skin immunity. Thus, the inoculation with HSV-1 or VACV leads to severe, even life-threatening complications eczema herpeticum or eczema vaccinatum, caused by the uncontrolled spread of the individual viruses, respectively [41]. Eczema herpeticum represents a relatively common, unpleasant complication, while the risk of eczema vaccinatum has led to the exclusion of atopic individuals from the preventive vaccination schemes against smallpox. With the increasing incidence of the atopic condition, studies on the pathogenesis, treatment and prevention of eczema herpeticum and vaccinatum remain important. Consequently, various animal models reproducing distinct aspects of the human AD have proved useful [41,78,79,80]. The time course and character of skin infection by WR-Luc described in this work were comparable to the effects of wt-WR observed in mock-sensitized [4], as well as in untreated Nc/Nga mice (unpublished results). In contrast, infection by WR-IRF3 led to the faster resolution of skin lesions and earlier virus clearance together with the induction of higher protective immunity against the lethal dose of wt-WR, the surrogate of smallpox. Our results thus suggest that such a replicating vaccination vector might be advantageous and safe even in atopic individuals.

## 5. Conclusions

In summary, we proved that WR-IRF3 overcomes the block in type I IFN expression induced by VACV, modulates the expression of several cytokines and induces superior protective immune responses against a lethal poxvirus challenge in both atopic Nc/Nga and control Th2-skewed Balb/c mice. Additionally, the results may be informative for the design of other virus-based vaccines, as well as for the treatment of different viral infections.

## Figures and Tables

**Table 1:** Sequences of the primers.

Target	Forward Primer 5′-3′	Reverse Primer 5′-3′
Mu GAPDH	CGG TGC TGA GTA TGT CGT GGA	GGC AGA AGG GGC GGA GAT GA
Mu IFNα ^1^	GCA ACC CTC CTA GAC TCA TTC T	CCA GCA GGG CGT CTT CCT
Mu IFNβ	GCA CTG GGT GGA ATG AGA CT	AGT GGA GAG CAG TTG AGG ACA
Mu IFNγ	TGG CAT AGA TGT GGA AGA AAA GAG	TGC AGG ATT TTC ATG TCA CCA
Mu IL-1β	TCC ATT GAG GTG GAG AGC TT	GGA TGA GGA CAT GAG CAC CT
Mu IL-12 ^2^	TGT CAA TCA CGC TAC CTC CTC	GTC CAG AGA CTG GAA TGA CCC
Mu IL-17A	GGA CTC TCC ACC GCA ATG AA	TTT CCC TCC GCA TTG ACA CA
Mu IL-4	CTT GGA AGC CCT ACA GAC GAG	GGA GAT GGA TGT GCC AAA CG
Mu IL-10	AGG CGC TGT CAT CGA TTT CTC	GCC TTG TAG ACA CCT TGG TCT T
Mu TGFβ1	CAC TGA TAC GCC TGA GTG GC	TCG AAA GCC CTG TAT TCC GT
Mu TGFβ3	GCA AGA ATC TGC CCA CAA GG	CCA TTG GGC TGA AAG GTG TG
Mu TNFα	GAT CGG TCC CCA AAG GGA TG	TGA GGG TCT GGG CCA TAG AA
Mu AIM2	AGG CAG TGG GAA CAA GAC AG	GAA ACC TTC CTG ACG CCA CC
Mu iNOS ^3^	ACG AGA CGG ATA GGC AGA GA	GCA CAT GCA AGG AAG GGA AC
Mu IRF-3	TAG GCT GGC TGT TGG AGA TGT	CCA GGT CTT CCA GCA GAC ACT

^1^ Primers detect the following gene products: Ifna1, 2, 4, 5, 6, 7, 9, 11, 12, 13, 14, 15, 16 and B [28]. ^2^ Primers detect the p35 subunit. ^3^ Primers detect all three transcription variants.

## Data Availability

Not applicable.

## Supplementary Materials

The following are available online at https://www.mdpi.com/article/10.3390/v13101986/s1, VACV IRF3 Supplements. Figure S1. Expression of mouse IRF-3 by WR-IRF3 in J774.G8 monocyte/macrophage cell line. Figure S2. Infection with WR-IRF3 increases mRNA levels of IL-1β in J774.G8 monocyte/macrophage cell line. Figure S3. Intranasal infection with WR-IRF3 supports higher mRNA levels of IL-1β in spleens of Nc/Nga and Balb/c mice. Figure S4. Intranasal immunization with WR-Luc and WR-IRF3 and protective immunity against a lethal challenge with wt-WR. Figure S5. Changes in cytokine mRNA levels in mouse skin lesions.

## References

[1] Artenstein A.W. (2008). New generation smallpox vaccines: A review of preclinical and clinical data. Rev. Med. Virol..

[2] Jacobs B.L., Langland J.O., Kibler K.V., Denzler K.L., White S.D., Holechek S.A., Wong S., Huynh T., Baskin C.R. (2009). Vaccinia virus vaccines: Past, present and future. Antiviral Res..

[3] Engler R.J., Kenner J., Leung D.Y. (2002). Smallpox vaccination: Risk considerations for patients with atopic dermatitis. J. Allergy Clin. Immunol..

[4] Knitlova J., Hajkova V., Voska L., Elsterova J., Obrova B., Melkova Z. (2014). Development of eczema vaccinatum in atopic mouse models and efficacy of MVA vaccination against lethal poxviral infection. PLoS ONE.

[5] Werfel T., Allam J.P., Biedermann T., Eyerich K., Gilles S., Guttman-Yassky E., Hoetzenecker W., Knol E., Simon H.U., Wollenberg A. (2016). Cellular and molecular immunologic mechanisms in patients with atopic dermatitis. J. Allergy Clin. Immunol..

[6] Mansouri Y., Guttman-Yassky E. (2015). Immune Pathways in Atopic Dermatitis, and Definition of Biomarkers through Broad and Targeted Therapeutics. J. Clin. Med..

[7] Wollenberg A., Wagner M., Gunther S., Towarowski A., Tuma E., Moderer M., Rothenfusser S., Wetzel S., Endres S., Hartmann G. (2002). Plasmacytoid dendritic cells: A new cutaneous dendritic cell subset with distinct role in inflammatory skin diseases. J. Investig. Dermatol..

[8] Kawakami Y., Tomimori Y., Yumoto K., Hasegawa S., Ando T., Tagaya Y., Crotty S., Kawakami T. (2009). Inhibition of NK cell activity by IL-17 allows vaccinia virus to induce severe skin lesions in a mouse model of eczema vaccinatum. J. Exp. Med..

[9] Howell M.D., Wollenberg A., Gallo R.L., Flaig M., Streib J.E., Wong C., Pavicic T., Boguniewicz M., Leung D.Y. (2006). Cathelicidin deficiency predisposes to eczema herpeticum. J. Allergy Clin. Immunol..

[10] Howell M.D., Gallo R.L., Boguniewicz M., Jones J.F., Wong C., Streib J.E., Leung D.Y. (2006). Cytokine milieu of atopic dermatitis skin subverts the innate immune response to vaccinia virus. Immunity.

[11] Ou L.S., Goleva E., Hall C., Leung D.Y. (2004). T regulatory cells in atopic dermatitis and subversion of their activity by superantigens. J. Allergy Clin. Immunol..

[12] Kohara Y., Tanabe K., Matsuoka K., Kanda N., Matsuda H., Karasuyama H., Yonekawa H. (2001). A major determinant quantitative-trait locus responsible for atopic dermatitis-like skin lesions in NC/Nga mice is located on Chromosome 9. Immunogenetics.

[13] Suto H., Matsuda H., Mitsuishi K., Hira K., Uchida T., Unno T., Ogawa H., Ra C. (1999). NC/Nga mice: A mouse model for atopic dermatitis. Int. Arch. Allergy Immunol..

[14] Smith G.L., Benfield C.T.O., Maluquer de Motes C., Mazzon M., Ember S.W.J., Ferguson B.J., Sumner R.P. (2013). Vaccinia virus immune evasion: Mechanisms, virulence and immunogenicity. J. Gen. Virol..

[15] Smith G.L., Talbot-Cooper C., Lu Y. (2018). How Does Vaccinia Virus Interfere With Interferon?. Adv. Virus Res..

[16] Unterholzner L., Sumner R.P., Baran M., Ren H., Mansur D.S., Bourke N.M., Randow F., Smith G.L., Bowie A.G. (2011). Vaccinia virus protein C6 is a virulence factor that binds TBK-1 adaptor proteins and inhibits activation of IRF3 and IRF7. PLoS Pathog..

[17] Ferguson B.J., Benfield C.T.O., Ren H., Lee V.H., Frazer G.L., Strnadova P., Sumner R.P., Smith G.L. (2013). Vaccinia virus protein N2 is a nuclear IRF3 inhibitor that promotes virulence. J. Gen. Virol..

[18] Liskova J., Knitlova J., Honner R., Melkova Z. (2011). Apoptosis and necrosis in vaccinia virus-infected HeLa G and BSC-40 cells. Virus Res..

[19] Benfield C.T.O., Ren H., Lucas S.J., Bahsoun B., Smith G.L. (2013). Vaccinia virus protein K7 is a virulence factor that alters the acute immune response to infection. J. Gen. Virol..

[20] Mansur D.S., Maluquer de Motes C., Unterholzner L., Sumner R.P., Ferguson B.J., Ren H., Strnadova P., Bowie A.G., Smith G.L. (2013). Poxvirus targeting of E3 ligase beta-TrCP by molecular mimicry: A mechanism to inhibit NF-kappaB activation and promote immune evasion and virulence. PLoS Pathog..

[21] Kalbacova M., Spisakova M., Liskova J., Melkova Z. (2008). Lytic infection with vaccinia virus activates caspases in a Bcl-2-inhibitable manner. Virus Res..

[22] Melkova Z., Esteban M. (1994). Interferon-gamma severely inhibits DNA synthesis of vaccinia virus in a macrophage cell line. Virology.

[23] Kalbacova M., Vrbacky M., Humlova Z., Melkova Z. (2002). Protooncogene Bcl-2 induces apoptosis in several cell lines. Folia Biol. (Praha).

[24] Rodriguez J.F., Rodriguez D., Rodriguez J.R., McGowan E.B., Esteban M. (1988). Expression of the firefly luciferase gene in vaccinia virus: A highly sensitive gene marker to follow virus dissemination in tissues of infected animals. Proc. Natl. Acad. Sci. USA.

[25] Mackett M., Smith G.L., Moss B. (1984). General method for production and selection of infectious vaccinia virus recombinants expressing foreign genes. J. Virol..

[26] Spisakova M., Cizek Z., Melkova Z. (2009). Ethacrynic and alpha-lipoic acids inhibit vaccinia virus late gene expression. Antiviral Res..

[27] Joklik W.K. (1962). The preparation and characteristics of highly purified radioactively labelled poxvirus. Biochim. Biophys Acta.

[28] Castellaneta A., Yoshida O., Kimura S., Yokota S., Geller D.A., Murase N., Thomson A.W. (2014). Plasmacytoid dendritic cell-derived IFN-alpha promotes murine liver ischemia/reperfusion injury by induction of hepatocyte IRF-1. Hepatology.

[29] Cogswell J.P., Godlevski M.M., Wisely G.B., Clay W.C., Leesnitzer L.M., Ways J.P., Gray J.G. (1994). NF-kappa B regulates IL-1 beta transcription through a consensus NF-kappa B binding site and a nonconsensus CRE-like site. J. Immunol..

[30] Chattopadhyay S., Marques J.T., Yamashita M., Peters K.L., Smith K., Desai A., Williams B.R., Sen G.C. (2010). Viral apoptosis is induced by IRF-3-mediated activation of Bax. EMBO J..

[31] Gherardi M.M., Ramirez J.C., Rodriguez D., Rodriguez J.R., Sano G., Zavala F., Esteban M. (1999). IL-12 delivery from recombinant vaccinia virus attenuates the vector and enhances the cellular immune response against HIV-1 Env in a dose-dependent manner. J. Immunol..

[32] von Sonnenburg F., Perona P., Darsow U., Ring J., von Krempelhuber A., Vollmar J., Roesch S., Baedeker N., Kollaritsch H., Chaplin P. (2014). Safety and immunogenicity of modified vaccinia Ankara as a smallpox vaccine in people with atopic dermatitis. Vaccine.

[33] Greenberg R.N., Hurley M.Y., Dinh D.V., Mraz S., Vera J.G., von Bredow D., von Krempelhuber A., Roesch S., Virgin G., Arndtz-Wiedemann N. (2015). A Multicenter, Open-Label, Controlled Phase II Study to Evaluate Safety and Immunogenicity of MVA Smallpox Vaccine (IMVAMUNE) in 18-40 Year Old Subjects with Diagnosed Atopic Dermatitis. PLoS ONE.

[34] Pittman P.R., Hahn M., Lee H.S., Koca C., Samy N., Schmidt D., Hornung J., Weidenthaler H., Heery C.R., Meyer T.P.H. (2019). Phase 3 Efficacy Trial of Modified Vaccinia Ankara as a Vaccine against Smallpox. N. Engl. J. Med..

[35] Yokote H., Shinmura Y., Kanehara T., Maruno S., Kuranaga M., Matsui H., Hashizume S. (2014). Safety of attenuated smallpox vaccine LC16m8 in immunodeficient mice. Clin. Vaccine Immunol..

[36] Danon Y.L., Sutter G. (2015). Use of the LC16m8 Smallpox Vaccine in Immunocompromised Individuals Is Still Too Risky. Clin. Vaccine Immunol..

[37] Ahn J., Barber G.N. (2019). STING signaling and host defense against microbial infection. Exp. Mol. Med..

[38] Ni G., Ma Z., Damania B. (2018). cGAS and STING: At the intersection of DNA and RNA virus-sensing networks. PLoS Pathog..

[39] Glanz A., Chakravarty S., Varghese M., Kottapalli A., Fan S., Chakravarti R., Chattopadhyay S. (2021). Transcriptional and Non-Transcriptional Activation, Posttranslational Modifications, and Antiviral Functions of Interferon Regulatory Factor 3 and Viral Antagonism by the SARS-Coronavirus. Viruses.

[40] Servant M.J., ten Oever B., LePage C., Conti L., Gessani S., Julkunen I., Lin R., Hiscott J. (2001). Identification of distinct signaling pathways leading to the phosphorylation of interferon regulatory factor 3. J. Biol. Chem..

[41] Reed J.L., Scott D.E., Bray M. (2012). Eczema vaccinatum. Clin. Infect. Dis..

[42] Thanos D., Maniatis T. (1995). Virus induction of human IFN beta gene expression requires the assembly of an enhanceosome. Cell.

[43] Panne D., Maniatis T., Harrison S.C. (2007). An atomic model of the interferon-beta enhanceosome. Cell.

[44] Garoufalis E., Kwan I., Lin R., Mustafa A., Pepin N., Roulston A., Lacoste J., Hiscott J. (1994). Viral induction of the human beta interferon promoter: Modulation of transcription by NF-kappa B/rel proteins and interferon regulatory factors. J. Virol..

[45] Roth S., Rottach A., Lotz-Havla A.S., Laux V., Muschaweckh A., Gersting S.W., Muntau A.C., Hopfner K.P., Jin L., Vanness K. (2014). Rad50-CARD9 interactions link cytosolic DNA sensing to IL-1beta production. Nat. Immunol..

[46] Aarreberg L.D., Wilkins C., Ramos H.J., Green R., Davis M.A., Chow K., Gale M. (2018). Interleukin-1beta Signaling in Dendritic Cells Induces Antiviral Interferon Responses. mBio.

[47] Abe T., Barber G.N. (2014). Cytosolic-DNA-mediated, STING-dependent proinflammatory gene induction necessitates canonical NF-kappaB activation through TBK1. J. Virol..

[48] Yum S., Li M., Fang Y., Chen Z.J. (2021). TBK1 recruitment to STING activates both IRF3 and NF-kappaB that mediate immune defense against tumors and viral infections. Proc. Natl. Acad. Sci. USA.

[49] Fang R., Wang C., Jiang Q., Lv M., Gao P., Yu X., Mu P., Zhang R., Bi S., Feng J.M. (2017). NEMO-IKKbeta Are Essential for IRF3 and NF-kappaB Activation in the cGAS-STING Pathway. J. Immunol..

[50] Guarda G., Braun M., Staehli F., Tardivel A., Mattmann C., Forster I., Farlik M., Decker T., Du Pasquier R.A., Romero P. (2011). Type I interferon inhibits interleukin-1 production and inflammasome activation. Immunity.

[51] Castiglia V., Piersigilli A., Ebner F., Janos M., Goldmann O., Dambock U., Kroger A., Weiss S., Knapp S., Jamieson A.M. (2016). Type I Interferon Signaling Prevents IL-1beta-Driven Lethal Systemic Hyperinflammation during Invasive Bacterial Infection of Soft Tissue. Cell Host Microbe.

[52] Tarassishin L., Loudig O., Bauman A., Shafit-Zagardo B., Suh H.S., Lee S.C. (2011). Interferon regulatory factor 3 inhibits astrocyte inflammatory gene expression through suppression of the proinflammatory miR-155 and miR-155*. Glia.

[53] Kettle S., Alcami A., Khanna A., Ehret R., Jassoy C., Smith G.L. (1997). Vaccinia virus serpin B13R (SPI-2) inhibits interleukin-1beta-converting enzyme and protects virus-infected cells from TNF- and Fas-mediated apoptosis, but does not prevent IL-1beta-induced fever. J. Gen. Virol..

[54] Lopez-Castejon G., Brough D. (2011). Understanding the mechanism of IL-1beta secretion. Cytokine Growth Factor Rev..

[55] Hornung V., Ablasser A., Charrel-Dennis M., Bauernfeind F., Horvath G., Caffrey D.R., Latz E., Fitzgerald K.A. (2009). AIM2 recognizes cytosolic dsDNA and forms a caspase-1-activating inflammasome with ASC. Nature.

[56] Gerlic M., Faustin B., Postigo A., Yu E.C., Proell M., Gombosuren N., Krajewska M., Flynn R., Croft M., Way M. (2013). Vaccinia virus F1L protein promotes virulence by inhibiting inflammasome activation. Proc. Natl. Acad. Sci. USA.

[57] Isaacs A., Lindenmann J. (1957). Virus interference. I. The interferon. Proc. R Soc. Lond B Biol. Sci..

[58] Lee S.B., Melkova Z., Yan W., Williams B.R., Hovanessian A.G., Esteban M. (1993). The interferon-induced double-stranded RNA-activated human p68 protein kinase potently inhibits protein synthesis in cultured cells. Virology.

[59] Rivas C., Gil J., Melkova Z., Esteban M., Diaz-Guerra M. (1998). Vaccinia virus E3L protein is an inhibitor of the interferon (i.f.n.)-induced 2-5A synthetase enzyme. Virology.

[60] Rodriguez J.R., Rodriguez D., Esteban M. (1991). Interferon treatment inhibits early events in vaccinia virus gene expression in infected mice. Virology.

[61] Liu G., Zhai Q., Schaffner D.J., Wu A., Yohannes A., Robinson T.M., Maland M., Wells J., Voss T.G., Bailey C. (2004). Prevention of lethal respiratory vaccinia infections in mice with interferon-alpha and interferon-gamma. FEMS Immunol. Med. Microbiol..

[62] Green D.R., Ferguson T., Zitvogel L., Kroemer G. (2009). Immunogenic and tolerogenic cell death. Nat. Rev. Immunol..

[63] Kepp O., Senovilla L., Galluzzi L., Panaretakis T., Tesniere A., Schlemmer F., Madeo F., Zitvogel L., Kroemer G. (2009). Viral subversion of immunogenic cell death. Cell Cycle.

[64] Humlova Z., Vokurka M., Esteban M., Melkova Z. (2002). Vaccinia virus induces apoptosis of infected macrophages. J. Gen. Virol..

[65] Baixeras E., Cebrian A., Albar J.P., Salas J., Martinez A.C., Vinuela E., Revilla Y. (1998). Vaccinia virus-induced apoptosis in immature B lymphocytes: Role of cellular Bcl-2. Virus Res..

[66] Engelmayer J., Larsson M., Subklewe M., Chahroudi A., Cox W.I., Steinman R.M., Bhardwaj N. (1999). Vaccinia virus inhibits the maturation of human dendritic cells: A novel mechanism of immune evasion. J. Immunol..

[67] Heylbroeck C., Balachandran S., Servant M.J., DeLuca C., Barber G.N., Lin R., Hiscott J. (2000). The IRF-3 transcription factor mediates Sendai virus-induced apoptosis. J. Virol..

[68] Apelbaum A., Yarden G., Warszawski S., Harari D., Schreiber G. (2013). Type I interferons induce apoptosis by balancing cFLIP and caspase-8 independent of death ligands. Mol. Cell Biol..

[69] Vestergaard C., Yoneyama H., Murai M., Nakamura K., Tamaki K., Terashima Y., Imai T., Yoshie O., Irimura T., Mizutani H. (1999). Overproduction of Th2-specific chemokines in NC/Nga mice exhibiting atopic dermatitis-like lesions. J. Clin. Investig..

[70] Komastu T., Ireland D.D., Reiss C.S. (1998). IL-12 and viral infections. Cytokine Growth Factor Rev..

[71] Fujikura Y., Kudlackova P., Vokurka M., Krijt J., Melkova Z. (2009). The effect of nitric oxide on vaccinia virus-encoded ribonucleotide reductase. Nitric Oxide.

[72] Melkova Z., Esteban M. (1995). Inhibition of vaccinia virus DNA replication by inducible expression of nitric oxide synthase. J. Immunol..

[73] Karupiah G., Xie Q.W., Buller R.M., Nathan C., Duarte C., MacMicking J.D. (1993). Inhibition of viral replication by interferon-gamma-induced nitric oxide synthase. Science.

[74] Huang S., Hendriks W., Althage A., Hemmi S., Bluethmann H., Kamijo R., Vilcek J., Zinkernagel R.M., Aguet M. (1993). Immune response in mice that lack the interferon-gamma receptor. Science.

[75] Ma W.T., Yao X.T., Peng Q., Chen D.K. (2019). The protective and pathogenic roles of IL-17 in viral infections: Friend or foe?. Open Biol..

[76] Darling A.R., Freyschmidt E.J., Burton O.T., Koleoglou K.J., Oyoshi M.K., Oettgen H.C. (2014). IL-10 suppresses IL-17-mediated dermal inflammation and reduces the systemic burden of Vaccinia virus in a mouse model of eczema vaccinatum. Clin. Immunol..

[77] Patera A.C., Pesnicak L., Bertin J., Cohen J.I. (2002). Interleukin 17 modulates the immune response to vaccinia virus infection. Virology.

[78] Shiohara T., Hayakawa J., Mizukawa Y. (2004). Animal models for atopic dermatitis: Are they relevant to human disease?. J. Dermatol. Sci..

[79] Achdout H., Lustig S., Israely T., Erez N., Politi B., Tamir H., Israeli O., Waner T., Melamed S., Paran N. (2017). Induction, treatment and prevention of eczema vaccinatum in atopic dermatitis mouse models. Vaccine.

[80] Leung D.Y., Gao P.S., Grigoryev D.N., Rafaels N.M., Streib J.E., Howell M.D., Taylor P.A., Boguniewicz M., Canniff J., Armstrong B. (2011). Human atopic dermatitis complicated by eczema herpeticum is associated with abnormalities in IFN-gamma response. J. Allergy Clin. Immunol..

