# Supplementary material for: Vaccinia Virus Expressing Interferon Regulatory Factor 3 Induces Higher Protective Immune Responses against Lethal Poxvirus Challenge in Atopic Organism
Source: Viruses. 2021 Oct 3;13(10):1986. doi: 10.3390/v13101986 (PMC8539567; doi:10.3390/v13101986)
Supplement: Supplementary file 1 [file viruses-13-01986-s001.zip › viruses-1385705-SI.pdf]

Vaccinia Virus Expressing Interferon Regulatory Factor 3 Induces Higher Protective Immune Responses against Lethal Poxvirus Challenge in Atopic Organism

Hana Pilna [1,2,&], Vera Hajkova [1,2,&], Jarmila Knitlova [1,#], Jana Liskova [1,#], Jana Elsterova[1,†], and Zora Melkova [1,2,*]

[1] Department of Immunology and Microbiology, First Faculty of Medicine, Charles University, Studnickova 7, 128 00 Prague 2, Czech Republic; pilna.hana@gmail.com (HP); gugpacenpo@seznam.cz (VH); jarmila.knitlova@gmail.com (JK); janefox@biolog.cz (JL); elsterova@paru.cas.cz (JE); zmelk@LF1.cuni.cz (ZM)
[2] BIOCEV, Biotechnology and Biomedicine Center of the Academy of Sciences and Charles University in Vestec, Průmyslová 595, 252 50 Vestec, Czech Republic
[*] Correspondence: zmelk@LF1.cuni.cz; Tel.: +420-22496 8507
[&] The authors contributed equally to this work
[#] Current affiliation: Department of Biomaterials and Tissue Engineering, Institute of Physiology, Czech Academy of Sciences, Videnska 1083, 142 20 Prague 4, Czech Republic
[†] Current affiliation: Institute of Parasitology, Biology Centre, Czech Academy of Sciences, Branisovska 31, 370 05 Ceske Budejovice, Czech Republic and Veterinary Research Institute, Hudcova 296/70, 621 00 Brno, Czech Republic

Supplements

Figure *S*1

(a)

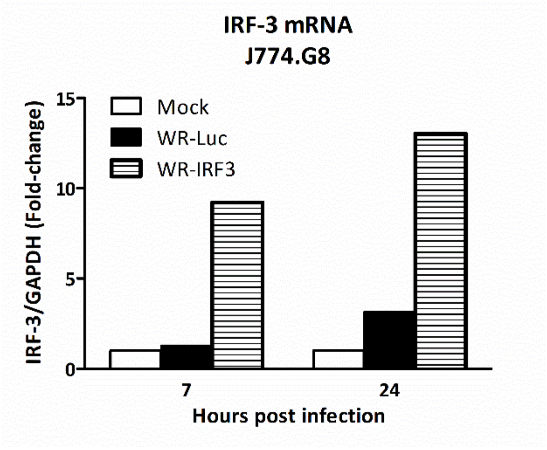

(b)

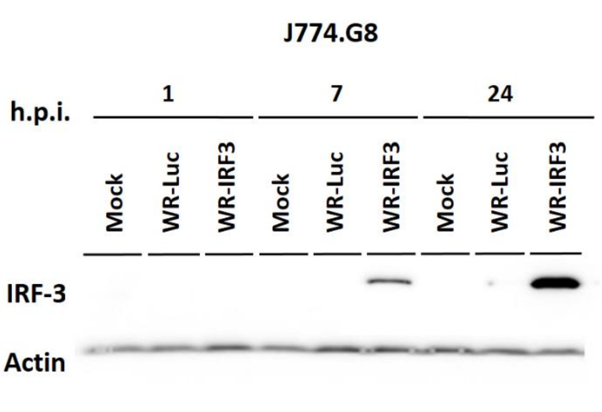

Figure S1. Expression of mouse IRF-3 by WR-IRF3 in J774.G8 monocyte/macrophage cell line. The cells were mock-infected or infected with WR-Luc or WR-IRF3 at M.O.I. 1. At individual time points, the cells were collected and used for further analysis. (**a**) 1-step RT-qPCR analysis at 24 h.p.i.. IRF-3 mRNA was normalized to GAPDH mRNA and expressed relatively as a fold-change compared to mock-infected cells. (**b**) Western blot analysis. The cell lysates were resolved by 12 % SDS-PAGE. IRF-3 and control *β*-actin were detected by western blot analysis and chemiluminescence. Mock – mock-infected cells; WR-Luc – cells infected with VACV expressing luciferase; WR-IRF3 – cells infected with VACV expressing Mu IRF-3.

Figure S2

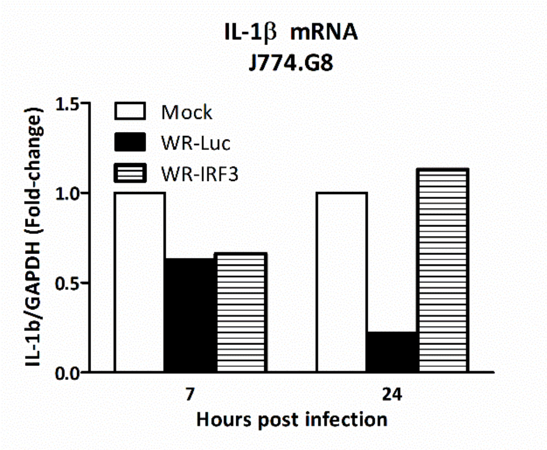

Figure S2. Infection with WR-IRF3 increases mRNA levels of IL-1β in J774.G8 monocyte/macrophage cell line. The cells were mock-infected or infected with WR-Luc or WR-IRF3 at M.O.I. 1. At indicated time points, the cells were collected and used for 1-step RT-qPCR analysis. IL-1β mRNA was normalized to GAPDH mRNA and expressed relatively as a fold-change compared to mock-infected cells. Mock – mock-infected cells; WR-Luc – cells infected with VACV expressing luciferase; WR-IRF3 – cells infected with VACV expressing Mu IRF-3.

Figure S3

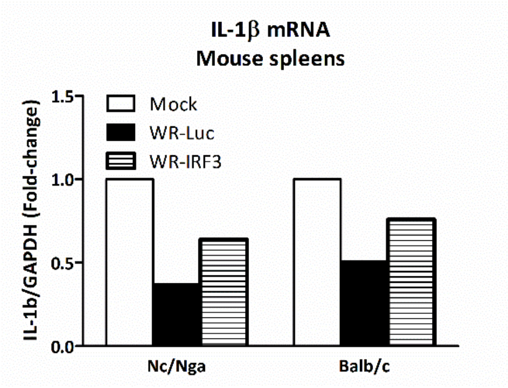

Figure S3. Intranasal infection with WR-IRF3 supports higher mRNA levels of IL-1β in spleens of Nc/Nga and Balb/c mice. The mice were i.n. mock-infected with PBS or infected with 4.37x 10^5 PFU of a purified stock of WR-Luc or WR-IRF3. At 3 days p.i., the mice were sacrificed and spleens were used for 1-step RT-qPCR analysis. IL-1β mRNA was normalized to GAPDH mRNA and expressed relatively as a fold-change compared to mock-infected samples. n = 3 mice/group. Mock – mock-infected mice; WR-Luc – mice infected with VACV expressing luciferase; WR-IRF3 – mice infected with VACV expressing Mu IRF-3.

Figure S4

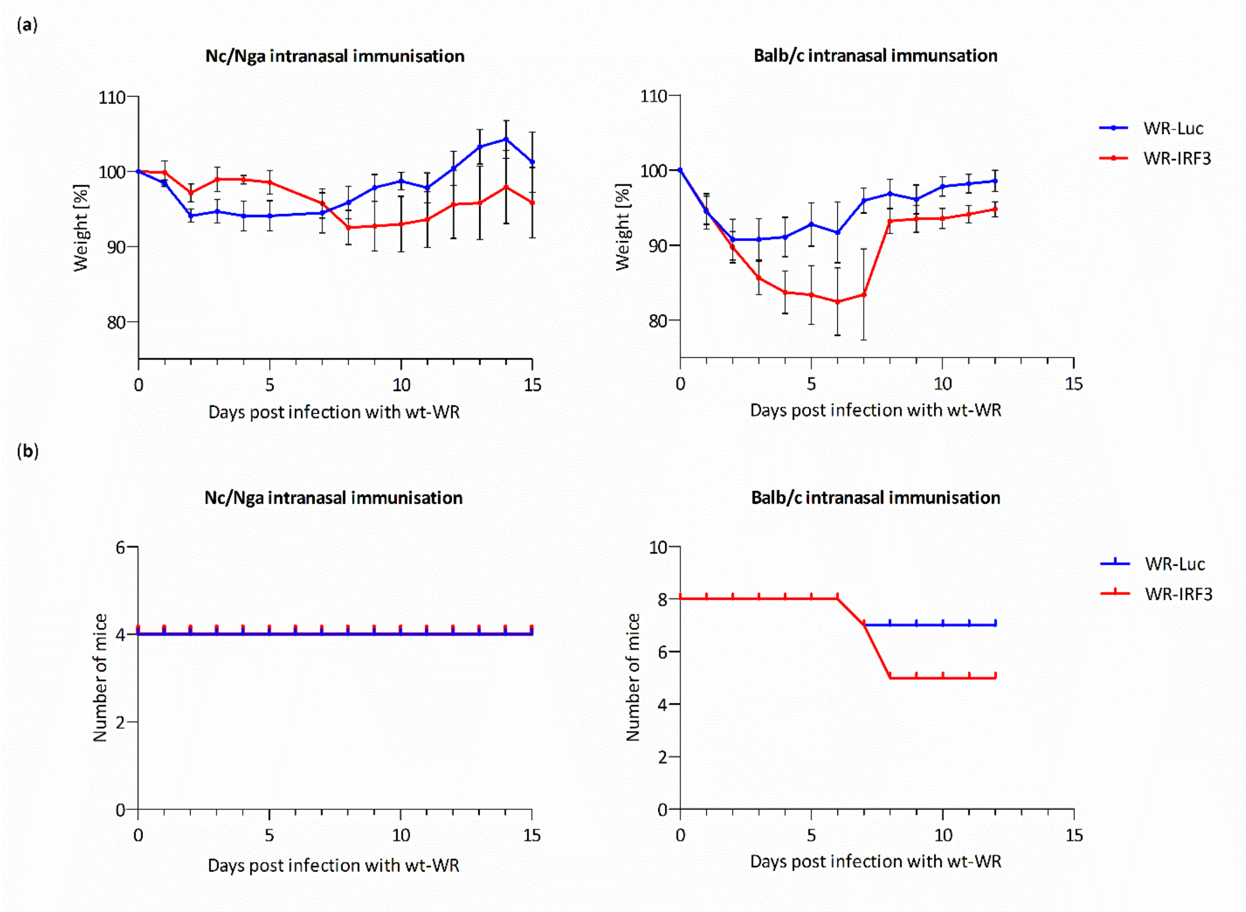

Figure S4. Intranasal immunization with WR-Luc and WR-IRF3 and protective immunity against a lethal challenge with wt-WR. Nc/Nga and Balb/c mice were immunized i.n. (10^4 PFU/mouse) with purified WR-Luc or WR-IRF3. 24 days later (day 0 in the graphs), they were infected i.n. with a lethal dose of wt-WR (10^5 PFU/mouse). Changes in body weight (**a**) and survival (**b**) of mice are presented. (**a**) Graphs represent means +/- S.E.M.

Figure S5

(a)

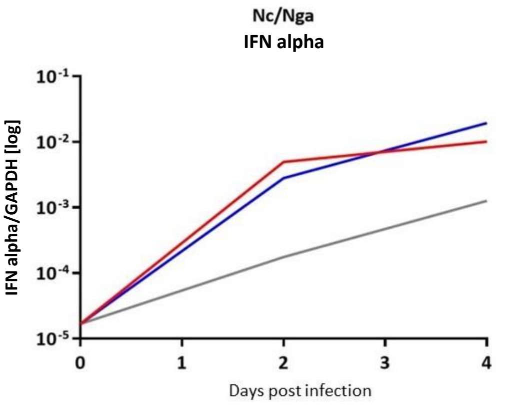

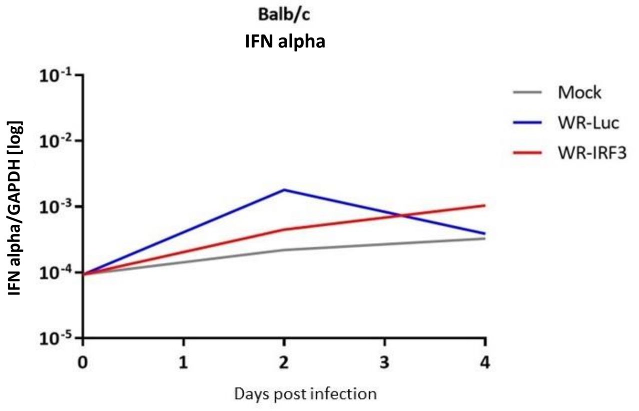

		GM	*÷ GM SD
T0		1,68E-05	19,25
Day 2	mock	1,76E-04	2,78
	WR-Luc	2,81E-03	1,84
	WR-IRF3	4,94E-03	2,14

		GM	*÷ GM SD
Day 4	mock	1,22E-03	5,58
	WR-Luc	1,94E-02	1,64
	WR-IRF3	1,01E-02	1,63

		GM	*÷ GM SD
T0		9,32E-05	3,17
Day 2	mock	2,20E-04	2,48
	WR-Luc	1,80E-03	2,70
	WR-IRF3	4,47E-04	1,76

		GM	*÷ GM SD
Day 4	mock	3,27E-04	1,43
	WR-Luc	3,88E-04	2,06
	WR-IRF3	1,04E-03	2,33

Fold change against T0		
d.p.i.	2	4
mock	10,48	72,75
WR-Luc	167,69	1156,87
WR-IRF3	294,84	602,19

Fold change against mock-infected mice		
d.p.i.	2	4
WR-Luc	16,00	15,90
WR-IRF3	28,13	8,28

Fold change against T0		
d.p.i.	2	4
mock	2,36	3,51
WR-Luc	19,28	4,16
WR-IRF3	4,80	11,21

Fold change against mock-infected mice		
d.p.i.	2	4
WR-Luc	8,19	1,19
WR-IRF3	2,04	3,19

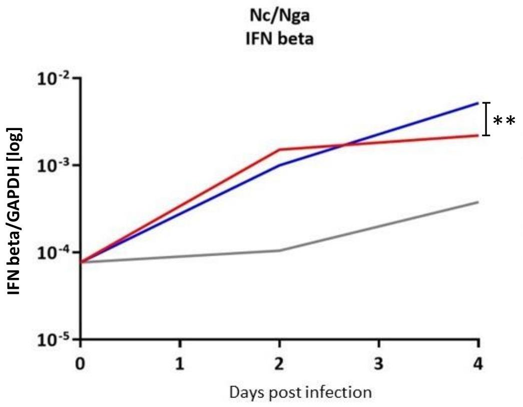

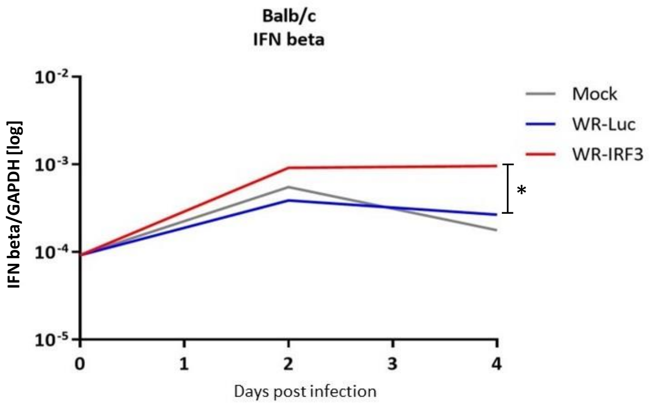

		GM	*÷ GM SD
T0		7,70E-05	2,00
Day 2	mock	1,05E-04	1,96
	WR-Luc	1,00E-03	2,74
	WR-IRF3	1,52E-03	2,41

		GM	*÷ GM SD
Day 4	mock	2,47E-04	2,83
	WR-Luc	5,19E-03	1,20
	WR-IRF3	2,21E-03	1,23

		GM	*÷ GM SD
T0		9,21E-05	1,37
Day 2	mock	5,50E-04	2,43
	WR-Luc	3,87E-04	3,92
	WR-IRF3	9,15E-04	1,60

		GM	*÷ GM SD
Day 4	mock	1,77E-04	1,22
	WR-Luc	2,67E-04	1,61
	WR-IRF3	9,55E-04	1,88

Fold change against T0		
d.p.i.	2	4
mock	1,36	3,21
WR-Luc	13,02	67,49
WR-IRF3	19,76	28,68

Fold change against mock-infected mice		
d.p.i.	2	4
WR-Luc	9,55	21,03
WR-IRF3	14,49	8,94

Fold change against T0		
d.p.i.	2	4
mock	5,98	1,93
WR-Luc	4,20	2,90
WR-IRF3	9,93	10,37

Fold change against mock-infected mice		
d.p.i.	2	4
WR-Luc	0,70	1,51
WR-IRF3	1,66	5,38

(b)

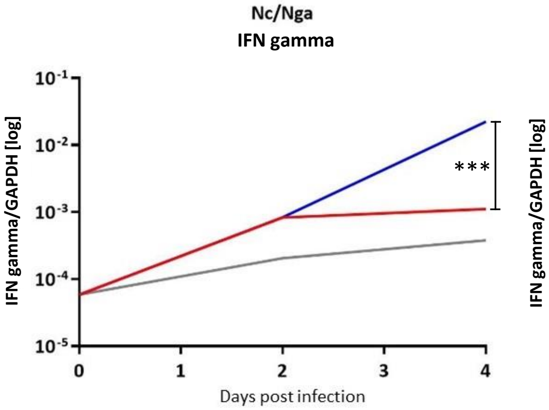

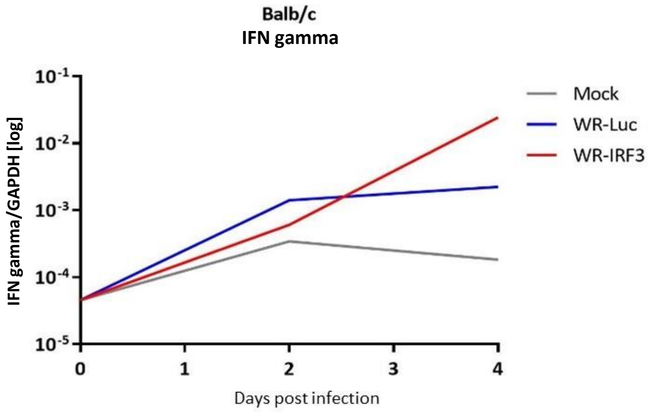

Nc/Nga IFN gamma

		GM	*÷ GM SD
T0		5,87E-05	1,39
Day 2	mock	2,06E-04	1,91
	WR-Luc	8,33E-04	2,26
	WR-IRF3	8,29E-04	1,67

		GM	*÷ GM SD
Day 4	mock	3,80E-04	2,54
	WR-Luc	2,23E-02	1,25
	WR-IRF3	1,11E-03	1,40

Fold change against T0

d.p.i.	2	4
mock	3,50	6,47
WR-Luc	14,19	380,36
WR-IRF3	14,11	18,89

Fold change against mock-infected mice

d.p.i.	2	4
WR-Luc	4,05	58,79
WR-IRF3	4,03	2,92

Balb/c IFN gamma

		GM	*÷ GM SD
T0		4,57E-05	1,26
Day 2	mock	3,42E-04	1,12
	WR-Luc	1,41E-03	3,02
	WR-IRF3	6,06E-04	2,02

		GM	*÷ GM SD
Day 4	mock	1,82E-04	1,22
	WR-Luc	2,23E-03	5,70
	WR-IRF3	2,43E-02	4,34

Fold change against T0

d.p.i.	2	4
mock	7,48	3,98
WR-Luc	30,86	48,73
WR-IRF3	13,26	532,47

Fold change against mock-infected mice

d.p.i.	2	4
WR-Luc	4,13	12,24
WR-IRF3	1,77	133,76

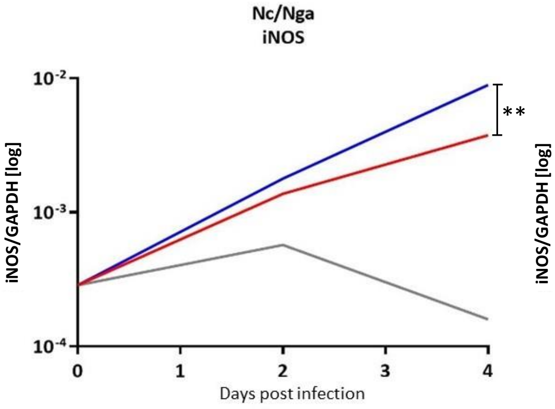

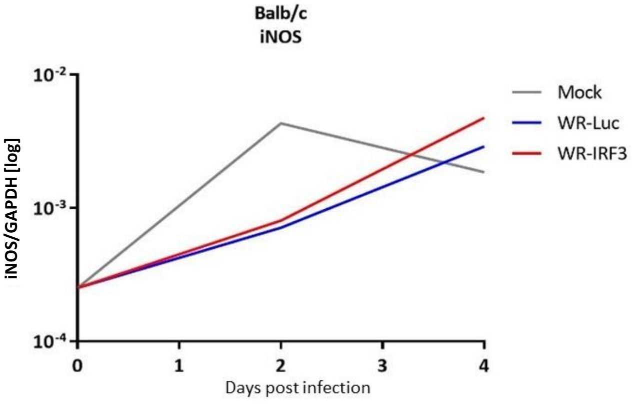

Nc/Nga iNOS

		GM	*÷ GM SD
T0		2,86E-04	3,79
Day 2	mock	5,69E-04	6,39
	WR-Luc	1,78E-03	1,35
	WR-IRF3	1,37E-03	3,75

		GM	*÷ GM SD
Day 4	mock	1,59E-04	2,41
	WR-Luc	8,88E-03	1,19
	WR-IRF3	3,75E-03	1,13

Fold change against T0

d.p.i.	2	4
mock	1,99	0,55
WR-Luc	6,22	31,02
WR-IRF3	4,79	13,09

Fold change against mock-infected mice

d.p.i.	2	4
WR-Luc	3,13	56,01
WR-IRF3	2,41	23,64

Balb/c iNOS

		GM	*÷ GM SD
T0		2,52E-04	2,37
Day 2	mock	4,32E-03	2,84
	WR-Luc	7,11E-04	4,91
	WR-IRF3	8,05E-04	1,80

		GM	*÷ GM SD
Day 4	mock	1,86E-03	1,18
	WR-Luc	2,90E-03	2,01
	WR-IRF3	4,75E-03	5,01

Fold change against T0

d.p.i.	2	4
mock	17,17	7,39
WR-Luc	2,83	11,54
WR-IRF3	3,20	18,88

Fold change against mock-infected mice

d.p.i.	2	4
WR-Luc	0,16	1,56
WR-IRF3	0,19	2,55

(c)

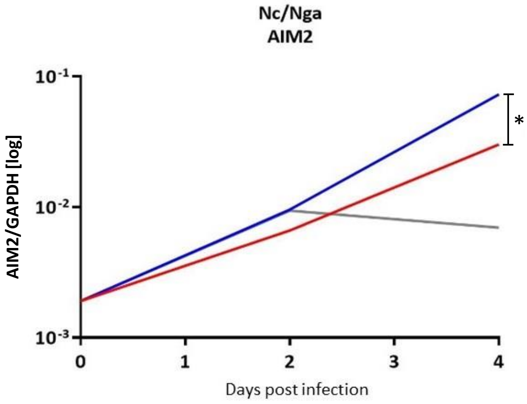

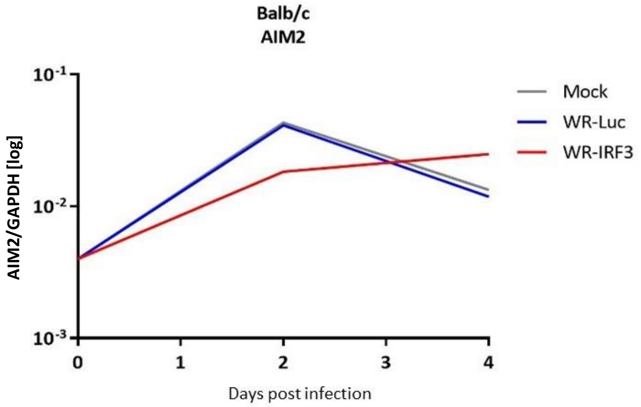

		GM	*÷ GM SD
T0		1,91E-03	3,64
Day 2	mock	9,40E-03	1,96
	WR-Luc	9,55E-03	1,65
	WR-IRF3	6,61E-03	2,24

		GM	*÷ GM SD
Day 4	mock	6,96E-03	2,87
	WR-Luc	7,29E-02	1,53
	WR-IRF3	3,01E-02	1,24

		GM	*÷ GM SD
T0		3,99E-03	1,33
Day 2	mock	4,21E-02	1,25
	WR-Luc	4,12E-02	2,53
	WR-IRF3	1,83E-02	1,55

		GM	*÷ GM SD
Day 4	mock	1,23E-02	1,29
	WR-Luc	1,18E-02	1,24
	WR-IRF3	2,49E-02	1,69

Fold change against T0

d.p.i.	2	4
mock	4,92	3,64
WR-Luc	5,00	38,19
WR-IRF3	3,46	15,75

Fold change against mock-infected mice

d.p.i.	2	4
WR-Luc	1,02	10,48
WR-IRF3	0,70	4,32

Fold change against T0

d.p.i.	2	4
mock	10,53	3,08
WR-Luc	10,32	2,96
WR-IRF3	4,59	6,25

Fold change against mock-infected mice

d.p.i.	2	4
WR-Luc	0,98	0,96
WR-IRF3	0,44	2,03

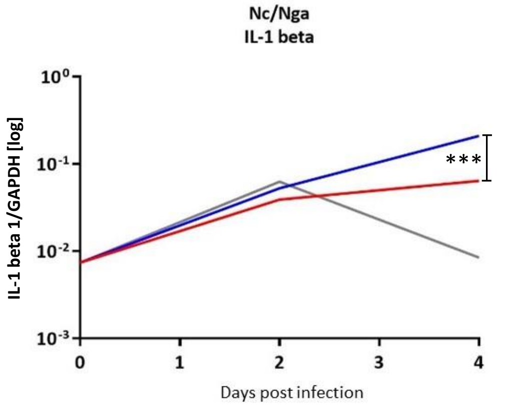

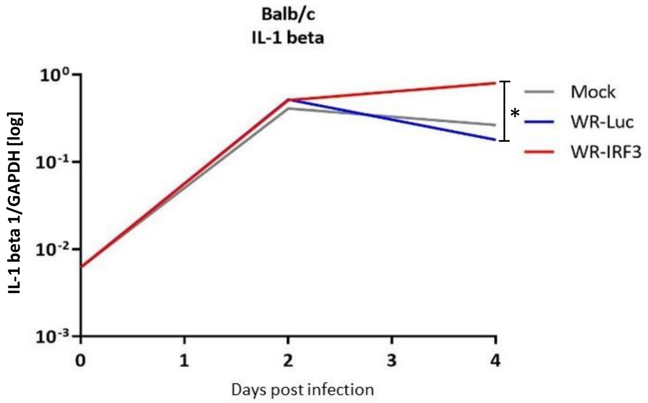

		GM	*÷ GM SD
T0		7,39E-03	3,55
Day 2	mock	6,26E-02	6,02
	WR-Luc	5,26E-02	2,81
	WR-IRF3	3,89E-02	2,32

		GM	*÷ GM SD
Day 4	mock	1,08E-02	1,11
	WR-Luc	2,10E-01	1,15
	WR-IRF3	6,40E-02	1,14

		GM	*÷ GM SD
T0		6,18E-03	1,98
Day 2	mock	4,10E-01	3,43
	WR-Luc	5,20E-01	6,65
	WR-IRF3	5,11E-01	2,16

		GM	*÷ GM SD
Day 4	mock	2,65E-01	2,29
	WR-Luc	1,79E-01	1,27
	WR-IRF3	8,00E-01	1,87

Fold change against T0

d.p.i.	2	4
mock	8,47	1,46
WR-Luc	7,12	28,40
WR-IRF3	5,27	8,65

Fold change against mock-infected mice

d.p.i.	2	4
WR-Luc	0,84	19,43
WR-IRF3	0,62	5,92

Fold change against T0

d.p.i.	2	4
mock	66,35	42,81
WR-Luc	84,10	28,92
WR-IRF3	82,62	129,39

Fold change against mock-infected mice

d.p.i.	2	4
WR-Luc	1,27	0,68
WR-IRF3	1,25	3,02

(d)

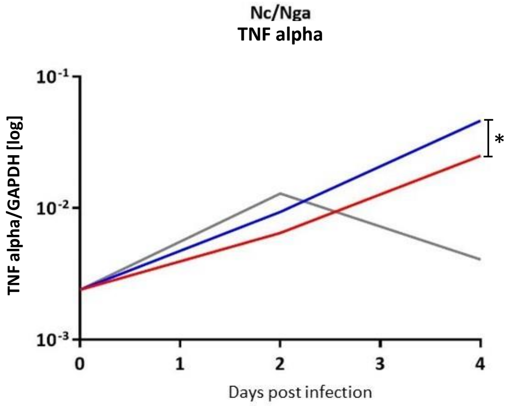

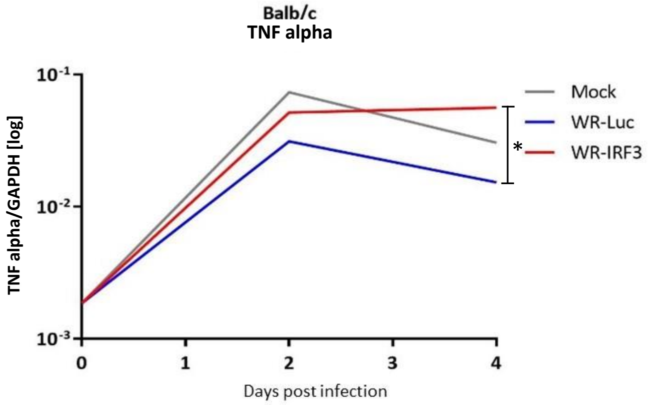

		GM	*÷ GM SD
T0		2,39E-03	2,14
Day 2	mock	1,29E-02	3,31
	WR-Luc	9,36E-03	1,26
	WR-IRF3	6,48E-03	2,04
Day 4	mock	4,06E-03	1,16
	WR-Luc	4,62E-02	1,18
	WR-IRF3	2,50E-02	1,24

Fold change against T0		
d.p.i.	2	4
mock	5,41	1,70
WR-Luc	3,92	19,32
WR-IRF3	2,71	10,48

Fold change against mock-infected mice		
d.p.i.	2	4
WR-Luc	0,72	11,37
WR-IRF3	0,50	6,17

		GM	*÷ GM SD
T0		1,86E-03	1,42
Day 2	mock	7,35E-02	4,61
	WR-Luc	3,12E-02	5,42
	WR-IRF3	5,15E-02	1,70
Day 4	mock	3,05E-02	2,57
	WR-Luc	1,53E-02	1,61
	WR-IRF3	5,62E-02	1,79

Fold change against T0		
d.p.i.	2	4
mock	39,47	16,37
WR-Luc	16,76	8,21
WR-IRF3	27,64	30,19

Fold change against mock-infected mice		
d.p.i.	2	4
WR-Luc	0,42	0,50
WR-IRF3	0,70	1,84

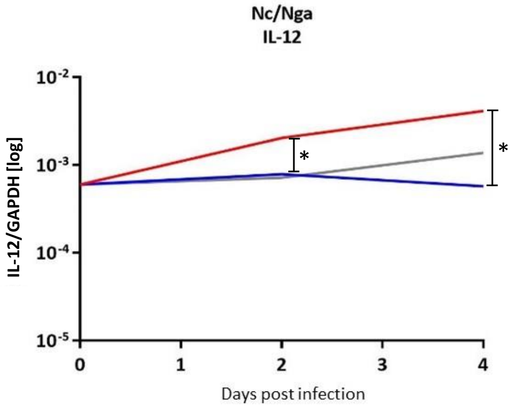

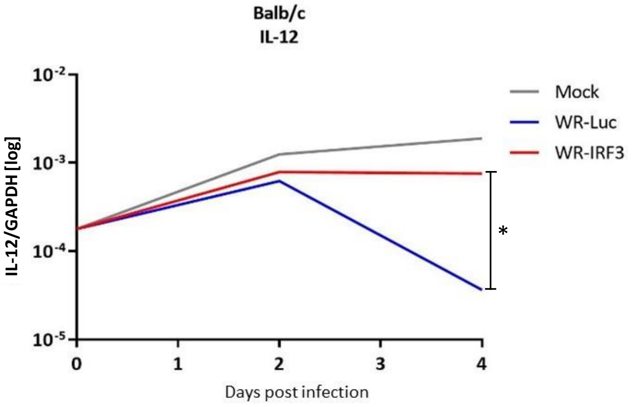

		GM	*÷ GM SD
T0		5,96E-04	3,25
Day 2	mock	7,15E-04	1,75
	WR-Luc	7,82E-04	1,57
	WR-IRF3	2,02E-03	1,39
Day 4	mock	1,37E-03	1,50
	WR-Luc	5,70E-04	1,95
	WR-IRF3	4,12E-03	1,94

Fold change against T0		
d.p.i.	2	4
mock	1,20	2,29
WR-Luc	1,31	0,96
WR-IRF3	3,40	6,91

Fold change against mock-infected mice		
d.p.i.	2	4
WR-Luc	1,09	0,42
WR-IRF3	2,83	3,02

		GM	*÷ GM SD
T0		1,78E-04	2,67
Day 2	mock	1,25E-03	1,70
	WR-Luc	6,22E-04	10,14
	WR-IRF3	7,88E-04	2,62
Day 4	mock	1,89E-03	1,52
	WR-Luc	3,65E-05	1,95
	WR-IRF3	7,58E-04	3,78

Fold change against T0		
d.p.i.	2	4
mock	7,01	10,64
WR-Luc	3,50	0,21
WR-IRF3	4,44	4,26

Fold change against mock-infected mice		
d.p.i.	2	4
WR-Luc	0,50	0,02
WR-IRF3	0,63	0,40

(e)

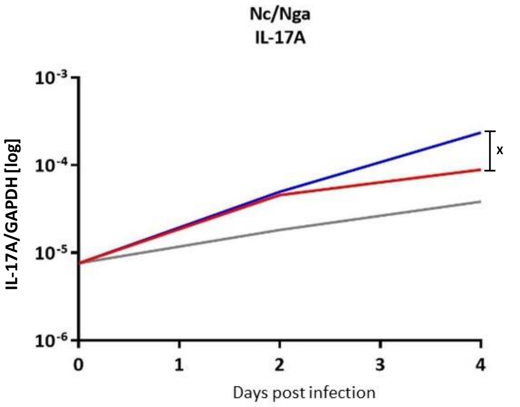

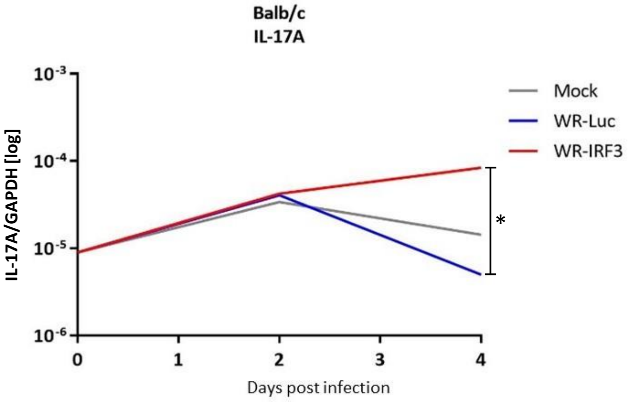

		GM	*÷ GM SD
T0		7,61E-06	1,88
Day 2	mock	1,83E-05	1,41
	WR-Luc	4,96E-05	2,55
	WR-IRF3	4,91E-05	2,62
Day 4	mock	1,64E-05	2,07
	WR-Luc	2,35E-04	1,80
	WR-IRF3	8,91E-05	1,61

Fold change against T0		
d.p.i.	2	4
mock	2,40	2,16
WR-Luc	6,52	30,82
WR-IRF3	6,45	11,71

Fold change against mock-infected mice		
d.p.i.	2	4
WR-Luc	2,71	14,30
WR-IRF3	2,49	5,43

		GM	*÷ GM SD
T0		5,08E-06	2,83
Day 2	mock	3,39E-05	1,97
	WR-Luc	4,06E-05	3,93
	WR-IRF3	4,24E-05	3,17
Day 4	mock	1,43E-05	4,30
	WR-Luc	5,00E-06	3,48
	WR-IRF3	8,38E-05	2,87

Fold change against T0		
d.p.i.	2	4
mock	6,67	2,81
WR-Luc	8,00	0,98
WR-IRF3	8,34	16,49

Fold change against mock-infected mice		
d.p.i.	2	4
WR-Luc	1,20	0,35
WR-IRF3	1,25	5,86
WR-Luc	1,20	0,35

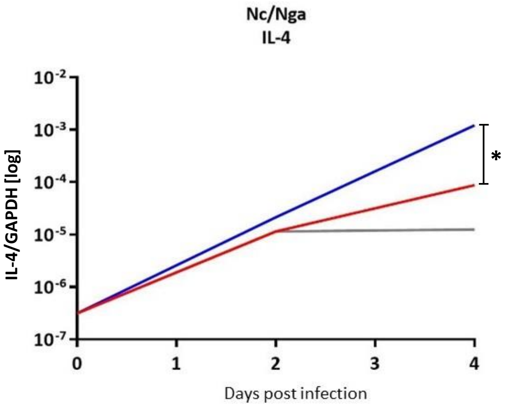

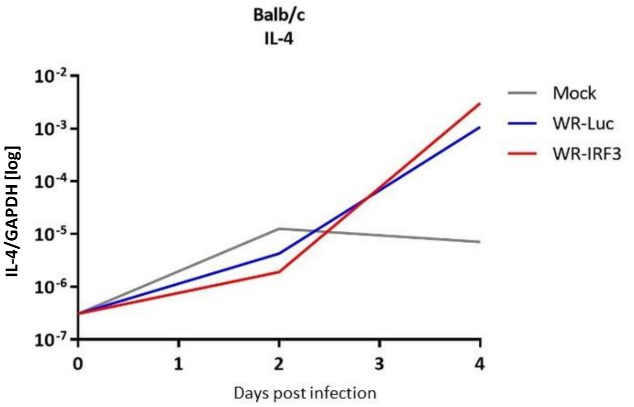

		GM	*÷ GM SD
T0		3,77E-07	2,23
Day 2	mock	1,15E-05	1,44
	WR-Luc	2,13E-05	1,56
	WR-IRF3	1,15E-05	2,03
Day 4	mock	1,25E-05	1,77
	WR-Luc	1,22E-03	1,61
	WR-IRF3	8,81E-05	3,51

Fold change against T0		
d.p.i.	2	4
mock	30,58	33,24
WR-Luc	56,63	3237,69
WR-IRF3	30,62	233,96

Fold change against mock-infected mice		
d.p.i.	2	4
WR-Luc	1,85	97,41
WR-IRF3	1,00	7,04

		GM	*÷ GM SD
T0		3,09E-07	28,50
Day 2	mock	1,27E-05	3,05
	WR-Luc	1,38E-06	16,59
	WR-IRF3	1,92E-06	36,01
Day 4	mock	7,11E-06	2,49
	WR-Luc	1,07E-03	1,99
	WR-IRF3	3,03E-03	2,53

Fold change against T0		
d.p.i.	2	4
mock	41,02	23,01
WR-Luc	4,47	3472,88
WR-IRF3	6,21	9810,77

Fold change against mock-infected mice		
d.p.i.	2	4
WR-Luc	0,34	150,92
WR-IRF3	0,15	426,34

(f)

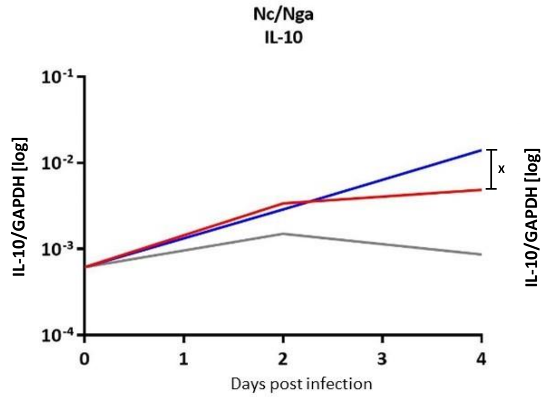

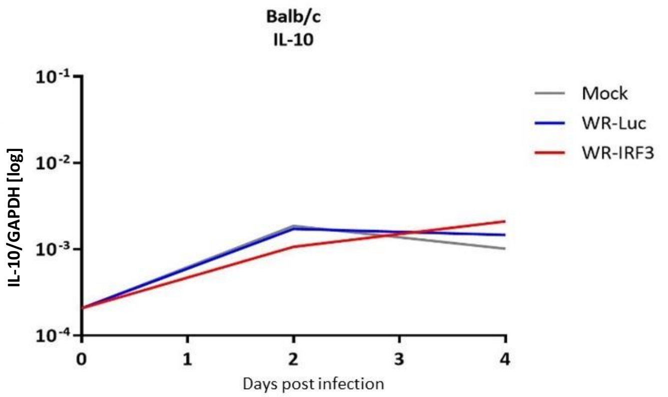

		GM	*÷ GM SD
T0		6,16E-04	1,99
Day 2	mock	1,50E-03	1,68
	WR-Luc	2,87E-03	1,68
	WR-IRF3	3,38E-03	3,86

		GM	*÷ GM SD
Day 4	mock	8,65E-04	1,38
	WR-Luc	1,40E-02	2,03
	WR-IRF3	4,86E-03	1,41

		GM	*÷ GM SD
T0		2,06E-04	1,19
Day 2	mock	1,86E-03	1,55
	WR-Luc	1,71E-03	4,71
	WR-IRF3	1,06E-03	1,40

		GM	*÷ GM SD
Day 4	mock	1,01E-03	1,21
	WR-Luc	1,46E-03	1,36
	WR-IRF3	2,10E-03	1,81

Fold change against T0

d.p.i.	2	4
mock	2,43	1,41
WR-Luc	4,65	22,66
WR-IRF3	5,49	7,89

Fold change against mock-infected mice

d.p.i.	2	4
WR-Luc	1,91	16,12
WR-IRF3	2,25	5,61

Fold change against T0

d.p.i.	2	4
mock	9,02	4,91
WR-Luc	8,29	7,08
WR-IRF3	5,16	10,19

Fold change against mock-infected mice

d.p.i.	2	4
WR-Luc	0,92	1,44
WR-IRF3	0,57	2,08

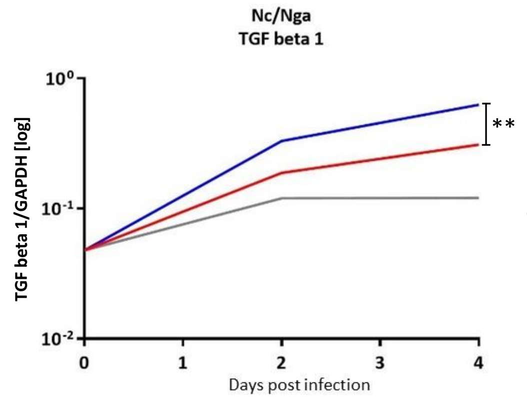

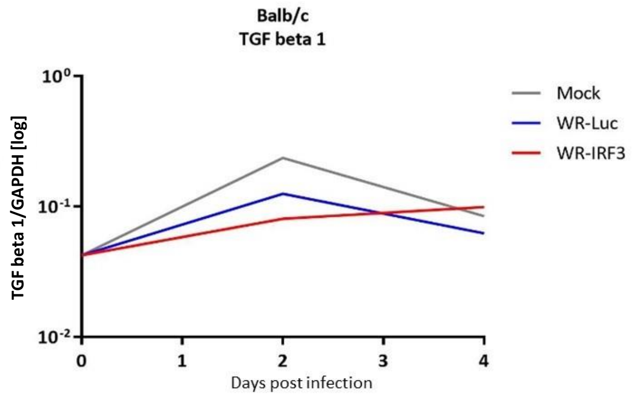

		GM	*÷ GM SD
T0		4,77E-02	1,35
Day 2	mock	1,20E-01	1,93
	WR-Luc	3,31E-01	1,58
	WR-IRF3	1,88E-01	3,42

		GM	*÷ GM SD
Day 4	mock	1,21E-01	3,73
	WR-Luc	6,28E-01	1,06
	WR-IRF3	3,10E-01	1,27

		GM	*÷ GM SD
T0		4,22E-02	1,49
Day 2	mock	2,36E-01	1,37
	WR-Luc	1,25E-01	5,30
	WR-IRF3	8,06E-02	1,52

		GM	*÷ GM SD
Day 4	mock	8,44E-02	3,93
	WR-Luc	6,23E-02	1,30
	WR-IRF3	9,92E-02	2,10

Fold change against T0

d.p.i.	2	4
mock	2,52	2,53
WR-Luc	6,93	13,15
WR-IRF3	3,95	6,49

Fold change against mock-infected mice

d.p.i.	2	4
WR-Luc	2,75	5,21
WR-IRF3	1,56	2,57

Fold change against T0

d.p.i.	2	4
mock	5,61	2,00
WR-Luc	2,97	1,48
WR-IRF3	1,91	2,35

Fold change against mock-infected mice

d.p.i.	2	4
WR-Luc	0,53	0,74
WR-IRF3	0,34	1,18

(g)

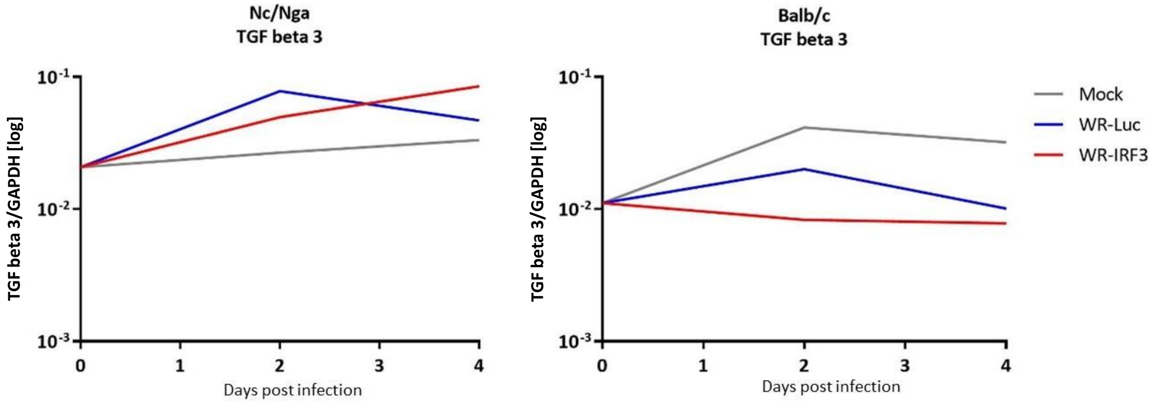

Nc/Nga

		GM	*÷ GM SD
T0		2,08E-02	1,74
Day 2	mock	2,68E-02	4,50
	WR-Luc	7,82E-02	1,85
	WR-IRF3	4,97E-02	3,05

		GM	*÷ GM SD
Day 4	mock	3,33E-02	3,50
	WR-Luc	4,69E-02	2,12
	WR-IRF3	8,50E-02	1,27

Fold change against T0		
d.p.i.	2	4
mock	1,29	1,60
WR-Luc	3,76	2,26
WR-IRF3	2,39	4,09

Fold change against mock-infected mice		
d.p.i.	2	4
WR-Luc	2,92	1,41
WR-IRF3	1,85	2,56

Balb/c

		GM	*÷ GM SD
T0		1,11E-02	2,25
Day 2	mock	4,15E-02	1,41
	WR-Luc	2,01E-02	7,84
	WR-IRF3	8,29E-03	1,52

		GM	*÷ GM SD
Day 4	mock	3,21E-02	1,77
	WR-Luc	1,01E-02	1,24
	WR-IRF3	7,81E-03	1,33

Fold change against T0		
d.p.i.	2	4
mock	3,74	2,89
WR-Luc	1,81	0,92
WR-IRF3	0,75	0,70

Fold change against mock-infected mice		
d.p.i.	2	4
WR-Luc	0,48	0,32
WR-IRF3	0,20	0,24

Figure S5. Changes in cytokine mRNA levels in mouse skin lesions. Nc/Nga and Balb/c mice were transdermally inoculated with PBS or 7.5×10^5 PFU/mouse of purified WR-Luc or WR-IRF3, sacrificed at indicated days p.i., and RNA from skin lesions used for 1-step RT-qPCR analysis. Cytokine mRNA was normalized to GAPDH mRNA and expressed as geometrical means; n = 3 mice/group. Statistically significant differences at ^{x} P < 0.1, * P < 0.05, ** P < 0.01, *** P < 0.001 (two-tailed unpaired Student t-test). GM *÷ GM SD and relative changes compared to time 0 and to mock-infected samples are listed in accompanying tables. Mock – mock-inoculated mice (administration of PBS); WR-Luc – mice inoculated with VACV expressing luciferase; WR-IRF3 – mice inoculated with VACV expressing Mu IRF-3; T0 – time of inoculation; d.p.i. – days post infection; GM – geometrical mean, GM SD – geometric standard deviation.
